# Modulation of Behavioral, Biochemical, Immunomodulatory, and Transcriptional Profiles by the Strain *Limosilactobacillus fermentum* U-21 in Combined Model of Parkinson’s Disease in Wistar Rats

**DOI:** 10.3390/ijms27010446

**Published:** 2025-12-31

**Authors:** Diana A. Reznikova, Olga B. Bekker, Alla V. Stavrovskaya, Dmitry N. Voronkov, Andrei A. Gerasimov, Anastasiia K. Pavlova, Ivan A. Potapov, Mikhail V. Ivanov, Veronika S. Letvinova, Maya V. Odorskaya, Dilara A. Mavletova, Aleksey A. Vatlin, Sergey N. Illarioshkin, Valery N. Danilenko

**Affiliations:** 1 Laboratory of Bacterial Genetics, Vavilov Institute of General Genetics, Russian Academy of Sciences, 119333 Moscow, Russia; reznikova.da@phystech.edu (D.A.R.); obbekker@mail.ru (O.B.B.); veranika.letvinava@gmail.com (V.S.L.); maya_epifanova@mail.ru (M.V.O.); mavletova@vigg.ru (D.A.M.); valerid@vigg.ru (V.N.D.); 2 Moscow Center for Advanced Studies, 20 Kulakova Str., 123592 Moscow, Russia; 3 Russian Center of Neurology and Neurosciences, 125367 Moscow, Russia; alla_stav@mail.ru (A.V.S.); voronkovdm@gmail.com (D.N.V.); drewgerasimov@gmail.com (A.A.G.); pav_nastasya@mail.ru (A.K.P.); potapov.i.a@neurology.ru (I.A.P.); kitazatot@yandex.ru (M.V.I.); snillario@gmail.com (S.N.I.)

**Keywords:** *Limosilactobacillus fermentum* U-21, pharmacobiotic, Parkinson’s disease, combined model, lactacystin

## Abstract

Since there is currently no cure for Parkinson’s disease, pharmacobiotic approaches based on gut microbiota—capable of producing pharmacologically active compounds—are under development. In this study, we propose LfU21, derived from the strain *Limosilactobacillus fermentum* U-21, as a candidate pharmacobiotic. To evaluate its efficacy, a combined LPS- and lactacystin (LAC)-induced Parkinson’s disease model was established in Wistar rats. Effects were assessed using behavioral, biochemical, immunomodulatory, and transcriptomic biomarkers. LfU21 administration reduced α-synuclein levels, altered motor performance in the “Rung ladder” test, and modulated *bdnf* gene expression in the right and left striata. Under LPS exposure, LfU21 prevented alterations in immune response markers, GSH levels, *drd2* and *bdnf* gene expression, and intestinal goblet cell counts. In LAC and LAC + LPS groups, LfU21 mitigated the rise in α-synuclein, the decline in *bdnf* expression, and behavioral deficits in the “Open Field” and “Rung ladder” tests, respectively. The multifunctional activity of LfU21 in a combined Parkinson’s disease model underscores its therapeutic potential and helps identify a target patient cohort for future clinical trials.

## 1. Introduction

Parkinson’s disease (PD) is one of the most common neurodegenerative disorders, with a particularly high prevalence in individuals over 65 years of age. Current estimates indicate that more than 6 million people worldwide are affected [1]. Recent studies also suggest that COVID-19 may exacerbate PD progression by inducing neuroinflammation through cytokine storms [2]. In the majority of cases, PD is not attributed to genetic mutations, which account for less than 15% of patients, underscoring the need for further research into its etiology [3,4,5,6,7]. A critical obstacle in PD management is its late diagnosis, which stems from the delayed onset of clinical symptoms and the absence of reliable biomarkers for early detection [8,9,10,11]. Clinically, motor symptoms of PD typically emerge only in later stages of the disease, following the loss of more than 50% of dopaminergic neurons. These motor manifestations are often preceded by a prolonged prodromal phase marked by progressive dysfunction of the autonomic nervous system. One prominent feature is enteric nervous system (ENS) impairment, which presents as gastrointestinal dysmotility and the accumulation of the pathological protein α-synuclein within ENS neurons [12]. According to a leading hypothesis in PD pathogenesis, misfolded α-synuclein may progressively spread from the peripheral nervous system to the central nervous system (CNS), including key regions such as the substantia nigra [13]. Intestinal inflammation has been proposed as a potential trigger for this pathological cascade [14], supported by evidence of elevated α-synuclein expression and increased epithelial barrier permeability in the colon [15,16].

The development of effective therapeutics for Parkinson’s disease (PD) is challenged by several critical factors. First, the disease involves a wide array of disorders (reflected in multiple biomarkers of damage) across various organs and systems. Second, existing and emerging synthetic or semi-synthetic drugs often lack the capacity to simultaneously address dysfunctions across these distinct biological domains. Third, many current animal models of PD demonstrate limited translational validity, creating significant barriers to extrapolating experimental data to human pathophysiology. Fourth, PD is characterized by considerable etiological and clinical heterogeneity, which complicates the careful selection of patient cohorts for clinical trials. In a recently published review, we proposed a new conceptual framework that may help address these challenges [8]. Our approach involves: (1) developing live biotherapeutic products derived from commensal bacteria (e.g., *Lactobacillus* spp.) isolated from healthy individuals, designed to exert a multi-target, potentially multifunctional effect; (2) constructing a functional architecture model of PD that delineates its key neurological, neuroendocrine, immunological, neuroinflammatory, biochemical, genetic, and epigenetic features; (3) selecting and combining animal models that accurately simulate the early, pre-symptomatic stages of PD; and (4) subsequently identifying patient cohorts for clinical trials that are appropriately matched to the multi-locus mechanism of action of the pharmacobiotic candidate.

The multifunctional connections between the enteric, central nervous, and immune systems position the gut as a strategic target for therapeutic interventions in neurodegenerative diseases. Consequently, the influence of probiotic bacteria and their metabolites on immune and neural regulatory processes is now an active area of research [8,17]. Oral administration of therapeutics based on them is essential, as it allows influencing the composition of the gut microbiome [18].

Classic toxic and neuroinflammatory models of Parkinson’s disease (PD) fail to replicate a key neuropathological hallmark of the human condition: the formation of Lewy bodies in CNS neurons. However, studies show that combined administration of neurotoxins and lactacystin can induce α-synuclein aggregates within substantia nigra neurons [19,20]. Therefore, we hypothesized that adding lactacystin to a neuroinflammatory model would similarly induce the formation of intraneuronal α-synuclein aggregates in neurons of the central and/or peripheral nervous system.

The strain *Limosilactobacillus fermentum* U-21 was investigated in this study as a candidate pharmacobiotic. It is deposited in the All-Russian Collection of Industrial Microorganisms (VKPM) under accession number B-12075. Its genome assembly is available in GenBank (NCBI: ASM286982v2). *L. fermentum U-21* possesses genes and biosynthetic pathways associated with potential neuromodulatory, immunomodulatory, and anti-inflammatory activity [21]. The strain has demonstrated significant in vitro and in vivo antioxidant activity. In an *E. coli* K-12 bioluminescence assay, its culture fluid inhibited paraquat-induced oxidative stress by 25% [22]. In the *Caenorhabditis elegans* model, *L. fermentum* U-21 increased the median lifespan of nematodes under oxidative stress [23]. Furthermore, in a rodent model of paraquat-induced PD, co-administration of *L. fermentum* U-21 prevented the degradation of dopaminergic neurons [23] and mitigated pathological changes in internal organs [24]. As a key step in developing a live biotherapeutic, we conducted a multi-omics analysis to identify the low-molecular-weight metabolites, proteins, and enzymes responsible for its bioactivity—the pharmacologically active ingredients (PAIs) [21,25,26].

This study evaluated a panel of biomarkers central to Parkinson’s disease (PD) pathogenesis. The analyzed endpoints included tyrosine hydroxylase and IBA1 in the brain; total and phosphorylated α-synuclein in intestinal myenteric plexus ganglia; striatal expression of the *ngf*, *drd2*, *bdnf*, and *trkB* genes; hepatic redox status markers (superoxide dismutase, SOD; catalase, CAT; reduced glutathione, GSH); liver cytokine levels (IL-6, IL-10, TNF); and performance in behavioral tests.

Research Objective: The primary objective was to evaluate the effects of the pharmacobiotic candidate LfU21 (*Limosilactobacillus fermentum* U-21) on these behavioral, biochemical, immunomodulatory, and transcriptional parameters in a combined LPS and lactacystin (LPS + LAC) model of PD in Wistar rats. A secondary goal was to utilize the resulting biomarker signatures to inform the stratification of patient cohorts for future clinical trials.

## 2. Results

### 2.1. Histological Study for Tyrosine Hydroxylase and IBA1

**Figure 1:**
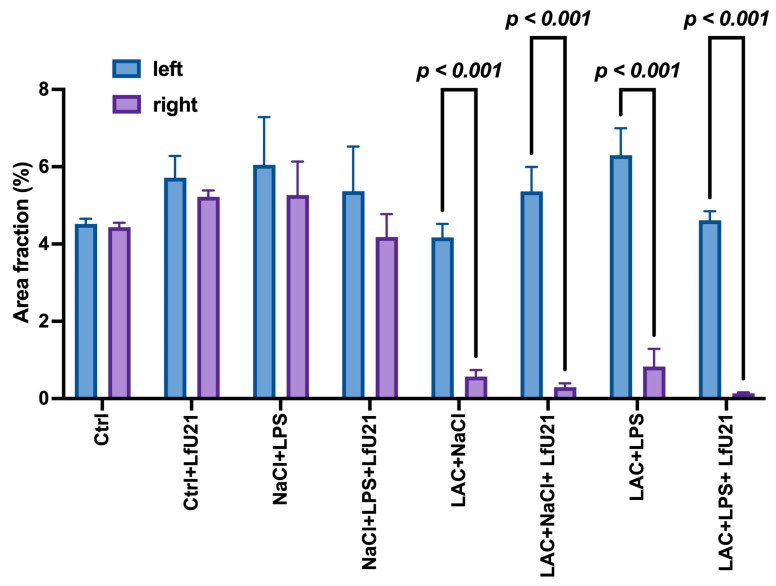
Immunohistochemical assessment of tyrosine hydroxylase (TH)-positive area in the substantia nigra. The left substantia nigra represents the intact control side, while the right substantia nigra shows the lesioned side. Data are presented as mean ± SEM. Statistical analysis was performed using three-way ANOVA.

**Figure 2:**
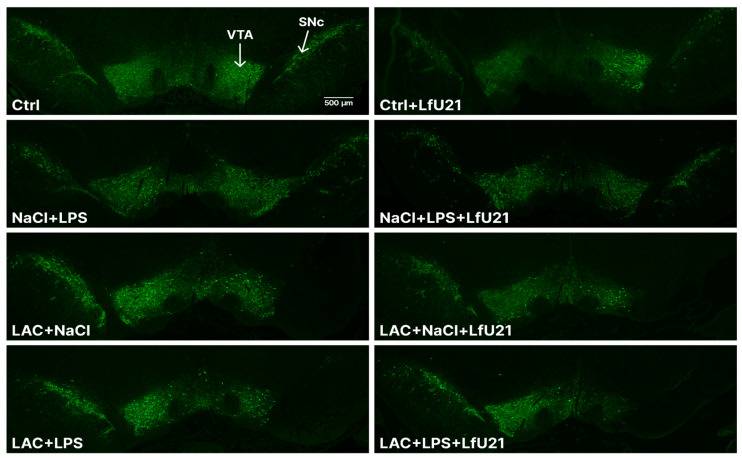
Changes in the intensity of staining for tyrosine hydroxylase in the substantia nigra pars compacta.

**Figure 3:**
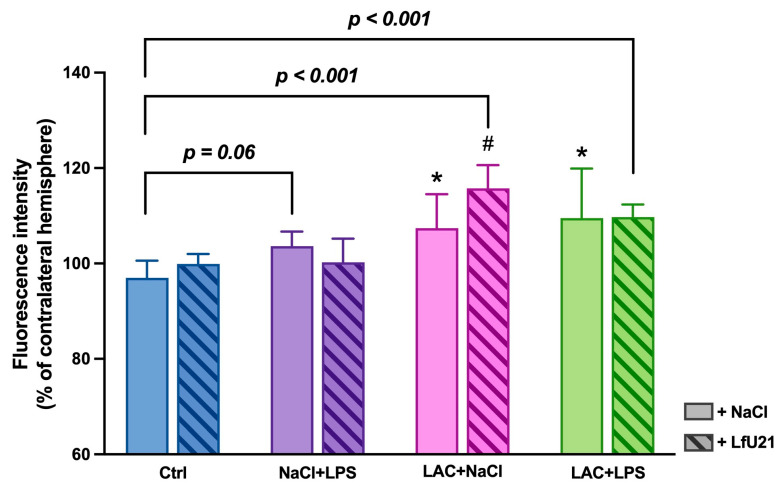
Effect of LfU21 on microglial activation following separate or combined administration of lactacystin (LAC) and lipopolysaccharide (LPS). IBA1 staining intensity is expressed as a percentage of the contralateral (left) substantia nigra (relative to the side of LAC or NaCl administration). Significant differences are indicated on the graphs: *—*p* < 0.05 vs. Ctrl; #—*p* < 0.05 vs. the corresponding groups not receiving LfU21. Statistical analysis was performed using three-way ANOVA. Data are presented as mean ± SEM.

**Figure 4:**
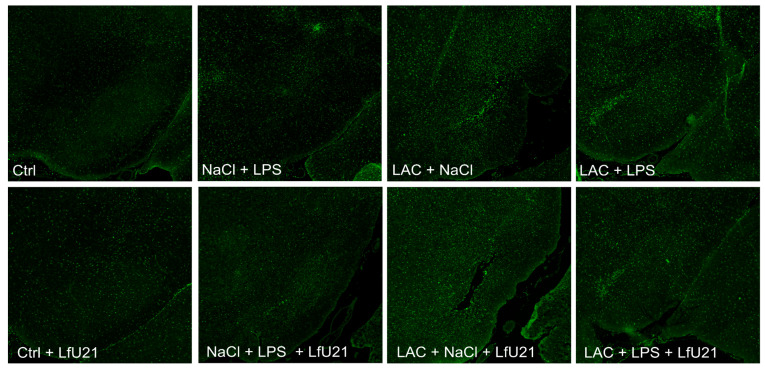
Changes in the intensity of staining for microglial marker IBA1 (right substantia nigra).

The integrity of the dopaminergic system and the extent of neuroinflammation were evaluated via immunohistochemical staining for tyrosine hydroxylase (a key enzyme in catecholamine synthesis) and the microglial marker IBA1. Intranigral administration of LAC, as well as combined exposure to LAC and LPS, resulted in a statistically significant reduction in the area of the right substantia nigra pars compacta (*p* < 0.05) (Figure 1 and Figure 2). Concurrently, these groups exhibited increased IBA1 immunostaining intensity (Figure 3 and Figure 4), indicative of microglial activation. In animals exposed to LPS alone, an increase in IBA1 staining intensity was observed at the level of a statistical trend (*p* = 0.0610) (Figure 3 and Figure 4). Administration of LfU21 did not significantly prevent either the neurodegenerative changes or the neuroinflammatory response in the substantia nigra pars compacta.

### 2.2. Transcriptional Biomarkers in the Rat Striatum

#### 2.2.1. Dopamine Receptor D2 Gene Expression

**Figure 5:**
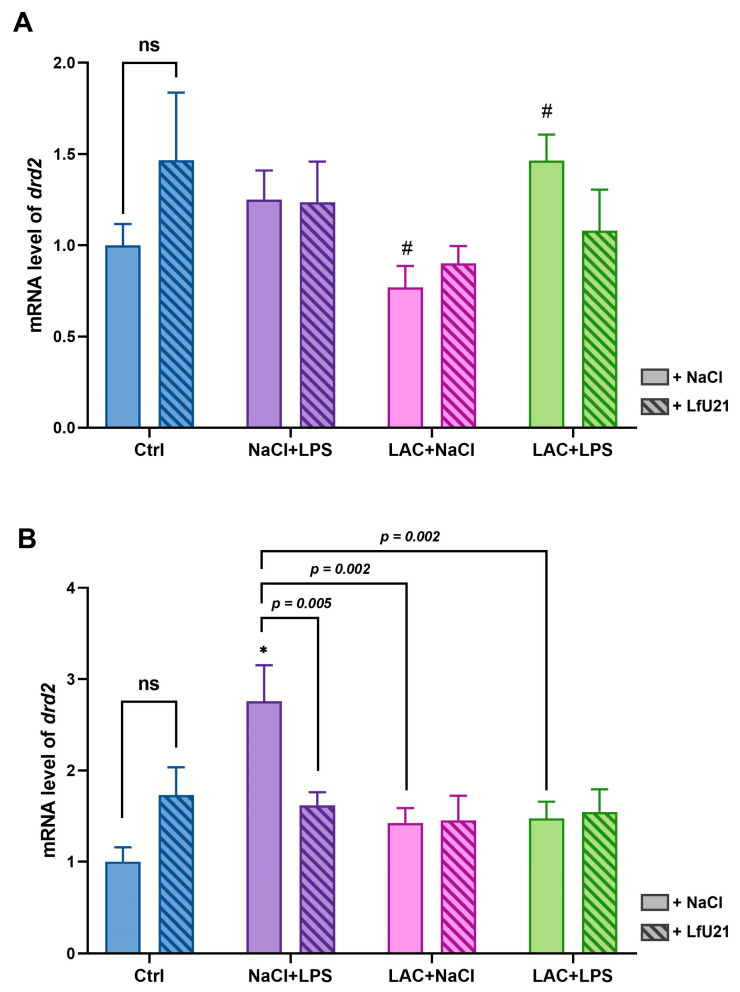
Expression of *drd2* mRNA in the striatum. (**A**) Left striatum. (**B**) Right striatum. Gene expression levels were normalized to *actb* cDNA. Data are presented as mean ± SEM from three biological replicates, each measured in triplicate. Expression in the untreated control group was set to 1. Statistical analysis was performed using three-way ANOVA. Significance indicators: ns, *p* ≥ 0.05; *—*p* < 0.001 vs. Ctrl; #—*p* < 0.05 vs. the correspondingly designated group.

The expression level of the dopamine D2 receptor gene (*drd2*) in the striatum was analyzed by RT-PCR (Figure 5). LfU21 administration alone did not significantly alter *drd2* expression in the right and left striatum compared to control animals. Intraperitoneal LPS administration caused a significant (~3-fold) increase in *drd2* expression in the right striatum (Figure 5B). This effect was corrected by co-administration of LfU21. Notably, the LPS-induced increase in the right striatum differed significantly from expression levels in both the lactacystin (LAC + NaCl) and the combined (LAC + LPS) PD model groups (Figure 5B). LAC administration alone did not change *drd2* expression in the right and left striatum. However, a significant difference in expression was observed in the left striatum between the LAC and the combined LAC + LPS groups (Figure 5A). A comparison of left and right striatum revealed significant interhemispheric differences in *drd2* expression within the NaCl + LPS (*p* < 0.05) and LAC + LPS (*p* < 0.05) groups (Figure S1).

#### 2.2.2. Nerve Growth Factor Gene Expression

**Figure 6:**
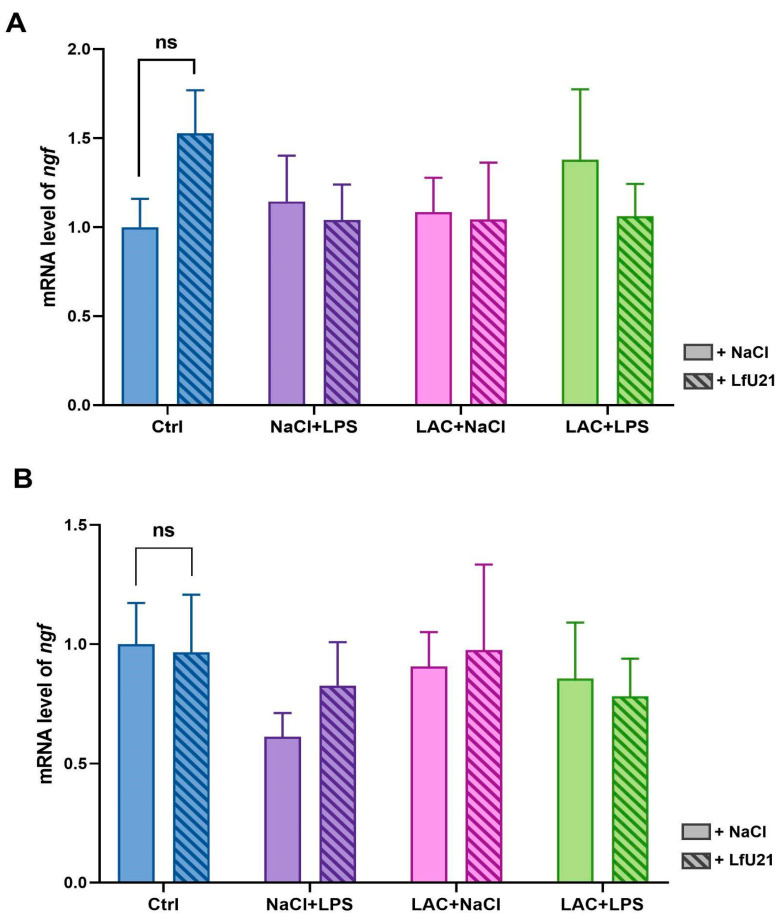
Expression of *ngf* mRNA in the striatum. (**A**) Left striatum. (**B**) Right striatum. Gene expression levels were normalized to *actb* cDNA. Data are presented as mean ± SEM from three biological replicates, each measured in triplicate. Expression in the untreated control group was set to 1. Statistical analysis was performed using three-way ANOVA (ns, *p* ≥ 0.05).

The expression level of the nerve growth factor (*ngf*) gene in the striatum was analyzed via RT-PCR (Figure 6). Administration of LfU21 alone did not significantly alter *ngf* expression in the right and left striatum compared to the control group (Figure 6). Intraperitoneal LPS injection resulted in a slight decrease in *ngf* expression in the right striatum (Figure 6B), whereas expression in the left striatum remained comparable to control levels (Figure 6A). Injection of LAC alone, as well as the combined administration of LPS and lactacystin, did not induce significant changes in *ngf* expression in the right and left striatum.

#### 2.2.3. Brain-Derived Neurotrophic Factor Gene Expression

**Figure 7:**
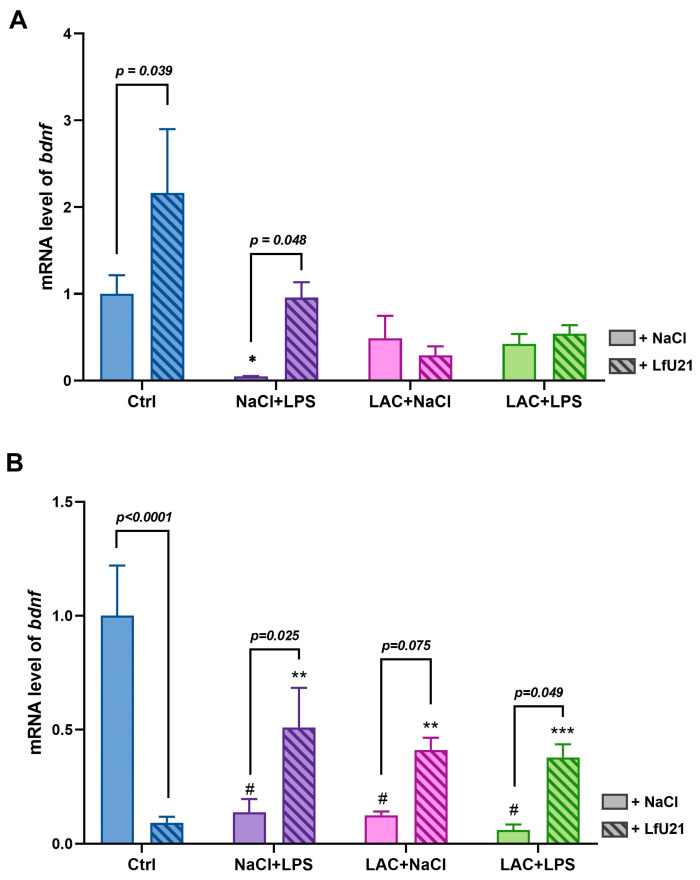
Expression of *bdnf* mRNA in the striatum. (**A**) Left striatum. (**B**) Right striatum. Gene expression levels were normalized to *actb* cDNA. Data are presented as mean ± SEM from three biological replicates, each measured in triplicate. Expression in the untreated control (Ctrl) group was set to 1. Statistical analysis was performed using three-way ANOVA. Significance indicators: *—*p* < 0.05, **—*p* < 0.01, ***—*p* < 0.001, and #—*p* < 0.0001 vs. Ctrl.

The expression level of the brain-derived neurotrophic factor gene (*bdnf*) in the striatum was analyzed by RT-PCR (Figure 7). LfU21 treatment alone significantly altered *bdnf* expression in the right and left striatum compared to the control. Intraperitoneal administration of LPS reduced *bdnf* expression in the right and left striatum; this decrease was attenuated by LfU21 co-administration. Injection of LAC, as well as the combined LAC + LPS treatment, resulted in a statistically significant decrease in *bdnf* expression specifically in the right striatum—the site of lactacystin administration (Figure 7B)—with no significant change observed in the left striatum (Figure 7A). Notably, LfU21 prevented this significant decrease in the combined (LAC + LPS) model in the right striatum (Figure 7B). A comparison of left and right striatum expression revealed significant interhemispheric differences in the Ctrl group (*p* < 0.001), with lower *bdnf* expression in the left striatum, and in the Ctrl + LfU21 group (*p* < 0.001), where expression was higher in the left striatum (Figure S2).

#### 2.2.4. Neuronal Receptor Tyrosine Kinase-2 Gene Expression

**Figure 8:**
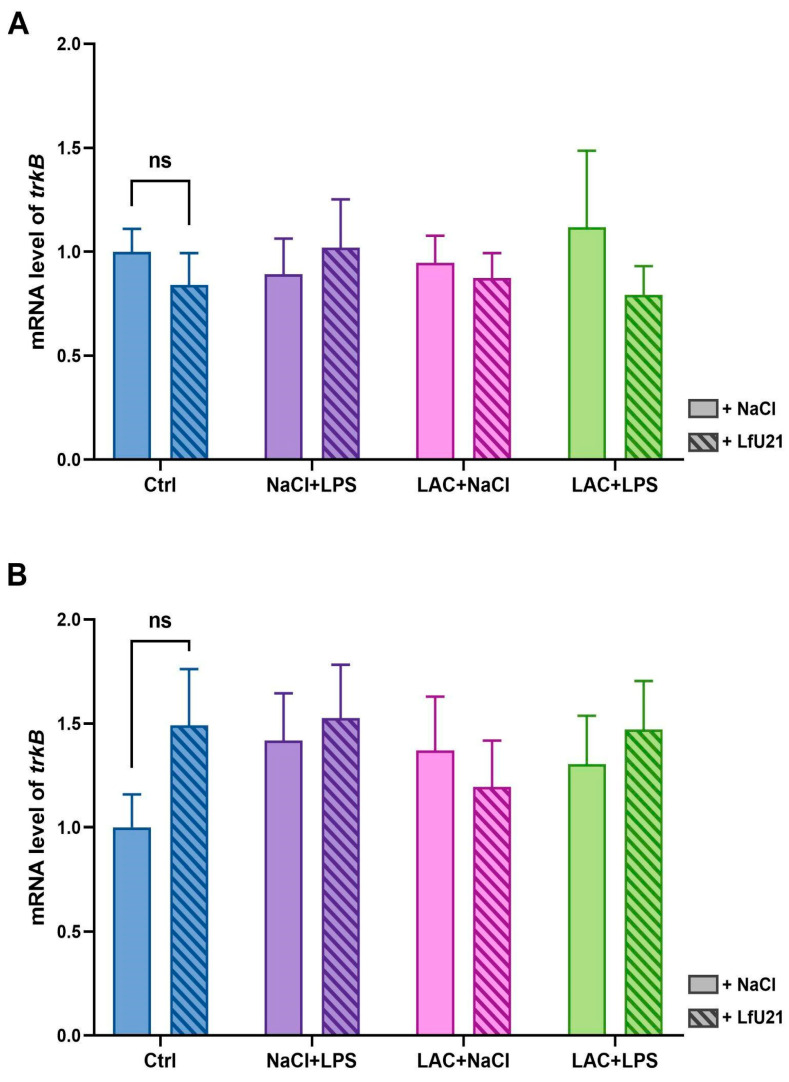
Expression of *trkB* mRNA in the striatum. (**A**) Left striatum. (**B**) Right striatum. Gene expression levels were normalized to *actb* cDNA. Data are presented as mean ± SEM from three biological replicates, each measured in triplicate. Expression in the untreated control group was set to 1. Statistical analysis was performed using three-way ANOVA (ns, *p* ≥ 0.05).

The expression level of the neuronal receptor tyrosine kinase-2 gene (*trkB*) was analyzed in the right and left striatum using RT-PCR (Figure 8). LfU21 administration had no statistically significant effect on *trkB* expression in any experimental group. Similarly, neither LAC nor LPS injection, alone or in combination, altered *trkB* gene expression.

### 2.3. Morphometric Study of the Small Intestine

#### 2.3.1. Immunofluorescence Staining for Total and Phosphorylated α-Synuclein in the Ganglia of the Myenteric Plexus of Wistar Rat Small Intestine

**Figure 9:**
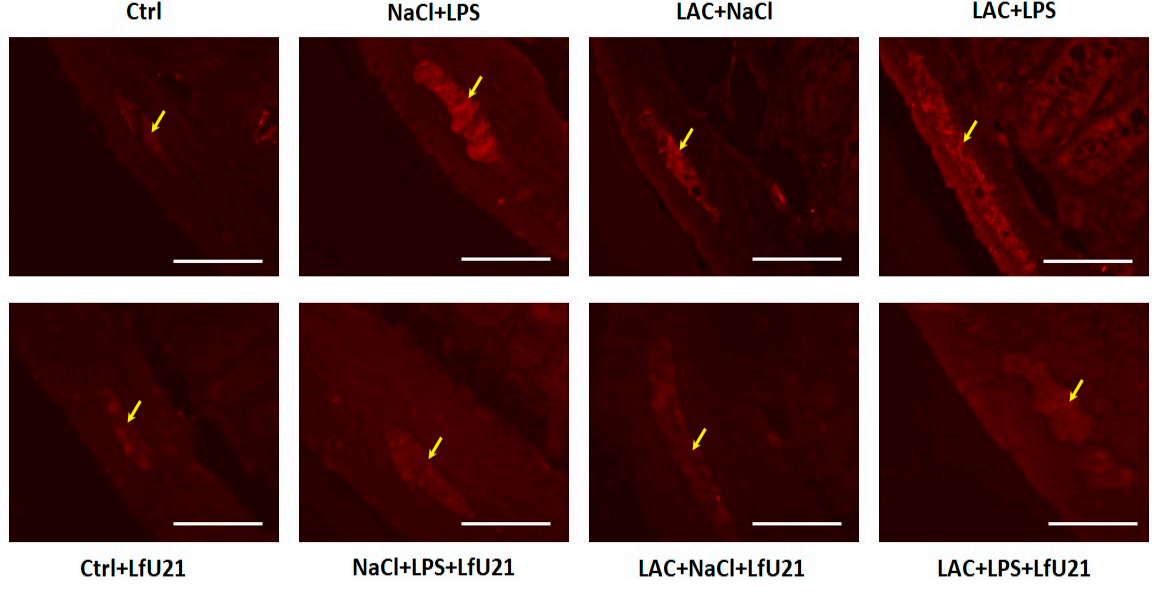
Representative immunofluorescence staining of total α-synuclein in the myenteric plexus ganglia (arrows) following LfU21 administration to lactacystin (LAC)−, lipopolysaccharide (LPS)−, and LAC + LPS-treated groups. Scale bar: 100 μm.

**Figure 10:**
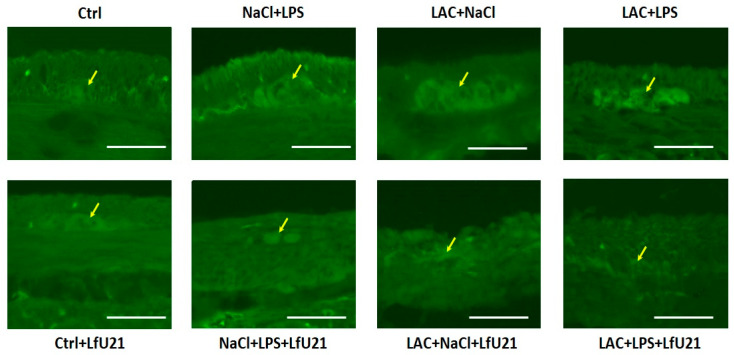
Representative immunofluorescence staining of phosphorylated α-synuclein in the myenteric plexus ganglia (arrows) following LfU21 administration to lactacystin (LAC)−, lipopolysaccharide (LPS)−, and LAC + LPS-treated groups. Scale bar: 50 μm.

**Figure 11:**
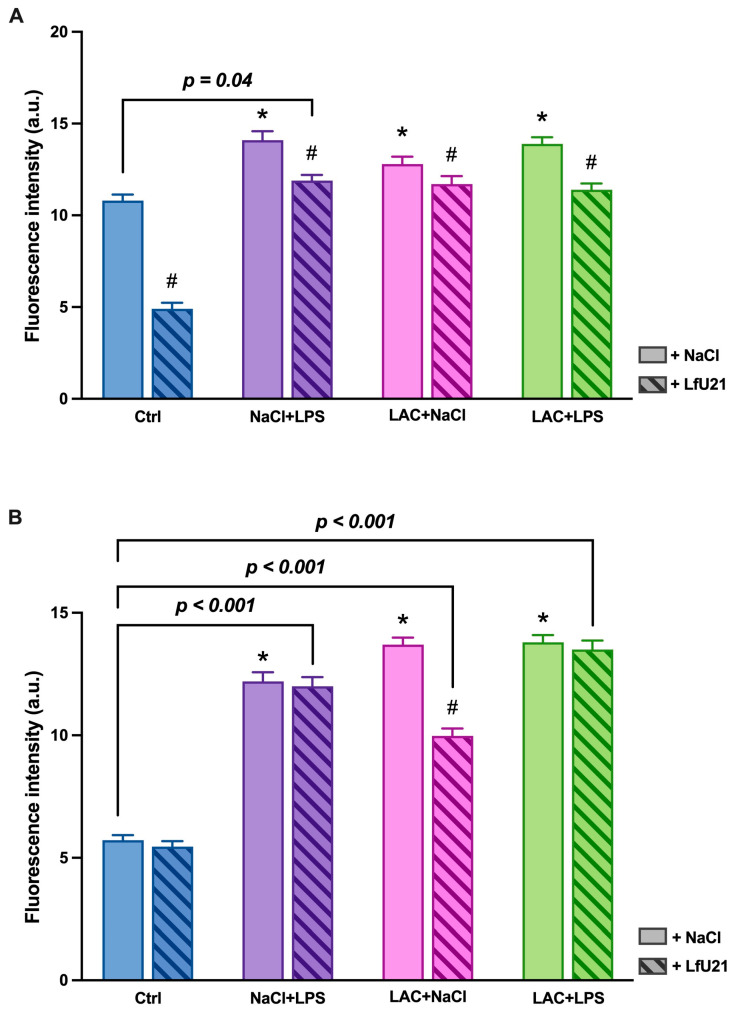
Intensity of immunofluorescence staining for total α-synuclein (**A**) and phosphorylated α-synuclein (**B**) in the ganglia of the myenteric plexus after administration of LfU21 to groups receiving LAC, LPS, and LAC + LPS. Data are presented as mean ± SEM. Statistical analysis was performed using three-way ANOVA. Significance indicators: *—*p* < 0.001 vs. Ctrl; #—*p* < 0.05 vs. the corresponding group without LfU21 treatment.

A statistically significant increase in the staining intensity of both total (Figure 9) and phosphorylated α-synuclein (Figure 10) was observed in the small intestine across all experimental groups compared to the control. Administration of LfU21 reduced the staining intensity for total α-synuclein in all groups and for phosphorylated α-synuclein specifically in the lactacystin-treated group (Figure 11).

#### 2.3.2. Assessment of Goblet Cell Counts in the Small Intestinal Epithelium of Rats

**Figure 12:**
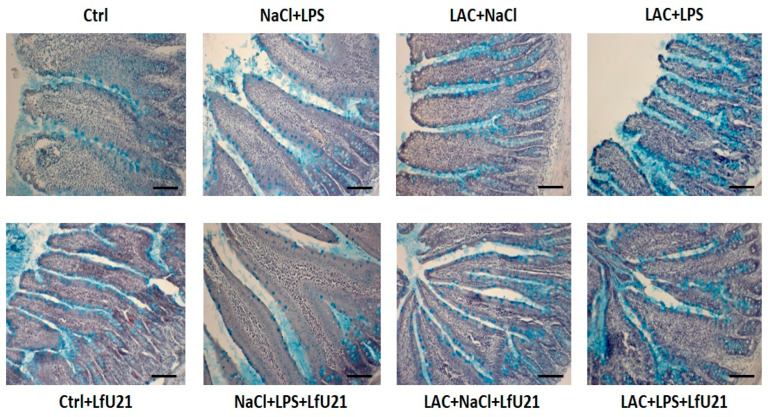
Representative images of goblet cells in the small intestinal epithelium. Sections from groups treated with LAC, LPS, or LAC + LPS, followed by LfU21 administration, were stained with Alcian blue. Scale bar: 100 μm.

**Figure 13:**
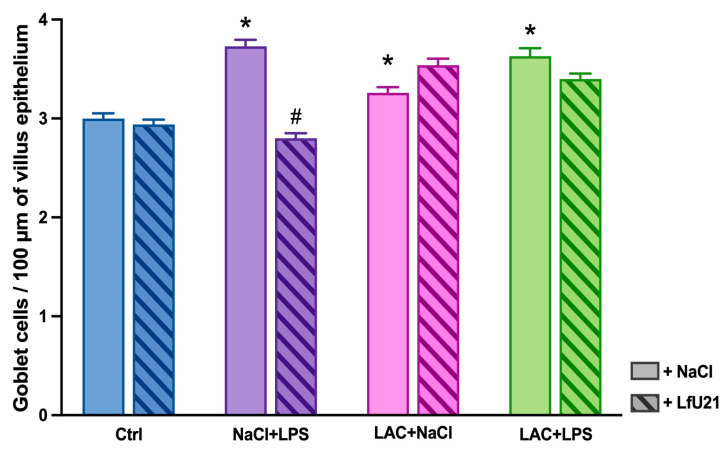
Quantification of goblet cells in the small intestinal epithelium. The number of goblet cells per 100 μm of villus length is shown for groups receiving lactacystin (LAC), lipopolysaccharide (LPS), or LAC + LPS, with or without LfU21 co-administration. Data are presented as mean ± SEM. Statistical analysis was performed using three-way ANOVA. Significance indicators: *—*p* < 0.001 vs. Ctrl; #—*p* < 0.05 vs. the corresponding group without LfU21.

A statistically significant increase in the number of goblet cells in the intestinal epithelium was observed both in the combined model and following the separate administration of LAC or LPS. The most pronounced increase occurred in the LPS-only group. LfU21 administration significantly reduced the number of goblet cells in this LPS-treated group (Figure 12 and Figure 13).

### 2.4. Investigation of Biochemical Markers in the Liver of Wistar Rats

**Figure 14:**
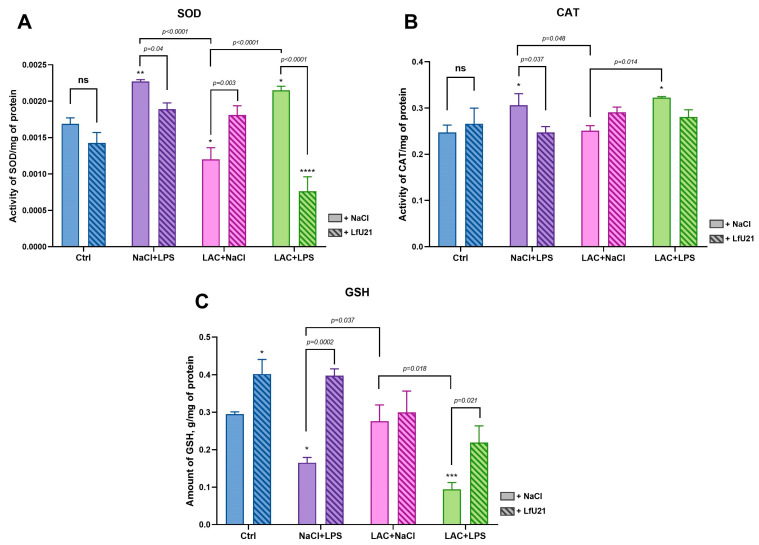
Hepatic antioxidant activity and glutathione levels in rats. (**A**) Total superoxide dismutase (SOD) activity. (**B**) Catalase (CAT) activity. (**C**) Reduced glutathione (GSH) concentration. Data are presented as mean ± SEM from three biological replicates. Statistical analysis was performed using three-way ANOVA. Significance indicators: ns, *p* ≥ 0.05; *—*p* < 0.05; **—*p* < 0.01; ***—*p* < 0.001; ****—*p* < 0.0001, all vs. Ctrl.

Under oxidative stress, the activity of superoxide dismutase (SOD)—which catalyzes the dismutation of the superoxide anion into oxygen and hydrogen peroxide—increases. Accordingly, SOD activity was elevated in the NaCl + LPS and LAC + LPS groups compared to the control, confirming an oxidative stress response (Figure 14A). LfU21 administration prevented this increase in SOD activity in the LAC + LPS model, resulting in levels significantly lower than in the corresponding untreated group and even below control values. In the lactacystin-only group (LAC + NaCl), SOD activity was reduced relative to control, and LfU21 normalized it to baseline. LfU21 alone did not alter SOD activity compared to the control.

Catalase (CAT), which decomposes hydrogen peroxide, also showed increased activity under oxidative stress, as seen in the LAC + LPS and NaCl + LPS groups (Figure 14B). LfU21 normalized CAT activity to control levels in the NaCl + LPS group, while its effect was less pronounced in the LAC + LPS group. LfU21 alone did not affect CAT activity.

Reduced glutathione (GSH) is oxidized by reactive oxygen species and is consequently depleted during oxidative stress (Figure 14C). A significant decrease in GSH was observed in the NaCl + LPS and LAC + LPS groups. LfU21 prevented this depletion in both the NaCl + LPS and LAC + LPS groups and, notably, increased hepatic GSH content above control levels.

### 2.5. Immunomodulatory Biomarkers in Liver of Wistar Rats

**Figure 15:**
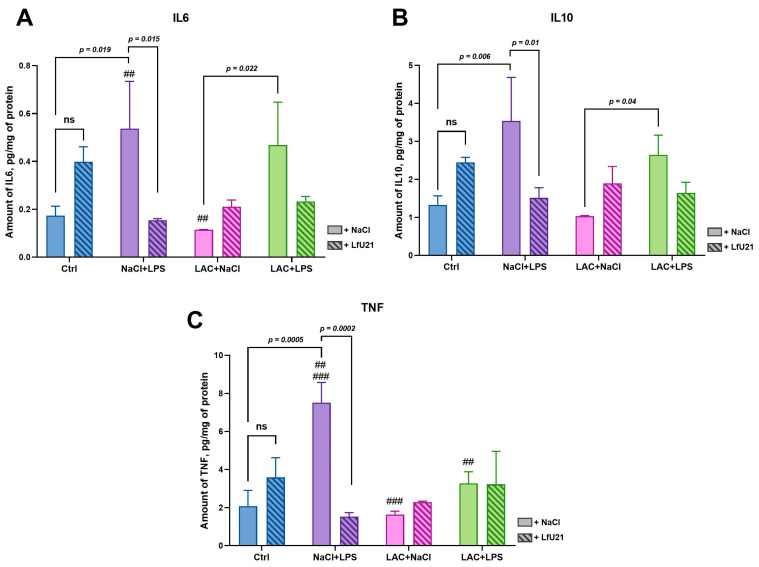
Hepatic cytokine levels in rats. (**A**) IL-6, (**B**) IL-10, and (**C**) TNF content. Data are presented as mean ± SEM from three biological replicates. Statistical analysis was performed using three-way ANOVA. Non-significant differences (ns) are defined as *p* ≥ 0.05. The # symbol indicates a significant difference compared to the correspondingly designated group: ##—*p* < 0.01, ###—*p* < 0.001.

The hepatic levels of the pro-inflammatory cytokines IL-6 and TNF and the anti-inflammatory cytokine IL-10 were assessed via enzyme-linked immunosorbent assay (ELISA) across experimental groups (Figure 15). LfU21 administration alone did not significantly alter the levels of IL-6, IL-10, or TNF.

Intraperitoneal injection of LPS significantly increased hepatic IL-6, IL-10, and TNF levels. This increase was prevented by co-administration of LfU21. In contrast, lactacystin (LAC) administration slightly reduced IL-6 and IL-10 levels, an effect that was also mitigated by LfU21. No significant cytokine activation was observed following combined LPS and LAC administration.

However, statistically significant differences in cytokine levels were detected between specific groups. For IL-6, levels differed between the NaCl + LPS and LAC + NaCl groups and between the LAC + NaCl and LAC + LPS groups. For IL-10, a difference was observed between the LAC + NaCl and LAC + LPS groups. For TNF, differences were noted between the NaCl + LPS and LAC + NaCl groups and between the NaCl + LPS and LAC + LPS groups (Figure 15).

### 2.6. Analysis of General Condition of Wistar Rats by Behavioral Tests

#### 2.6.1. Step Quality in the “Rung Ladder” Test

**Figure 16:**
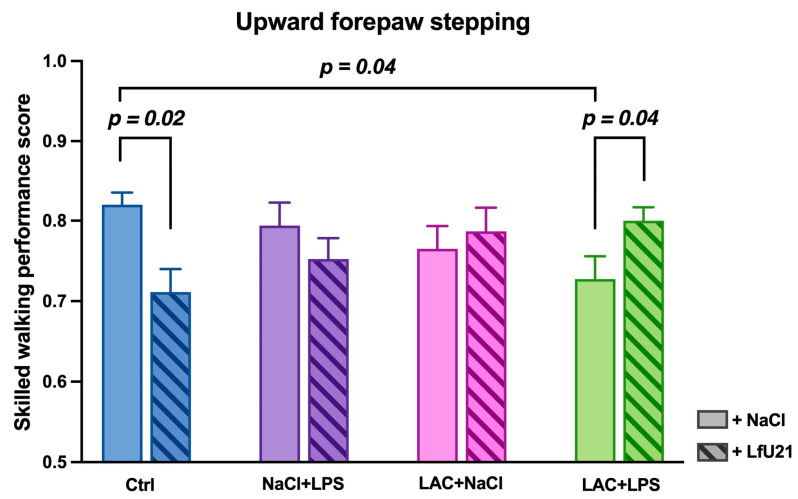
Effect of LfU21 on forelimb step quality in the Rung Ladder test. The quality of the forelimb step is shown for groups receiving lactacystin (LAC), lipopolysaccharide (LPS), or LAC + LPS, with or without LfU21 co-administration. Data are presented as mean ± SEM. Statistical analysis was performed using three-way ANOVA; significant differences are indicated on the graph.

The Rung Ladder test revealed that animals with the combined PD model (LAC + LPS) showed a significant decrease in forelimb step quality compared to controls (*p* = 0.04). LfU21 administration had a bidirectional effect: it prevented this decrease in step quality in the combined model group (LAC + LPS + LfU21; *p* = 0.04), while in control animals (Ctrl + LfU21), it paradoxically worsened performance (*p* = 0.02). In animals receiving LPS or LAC alone, LfU21 had no significant effect on forelimb step quality (*p* > 0.05) (Figure 16).

For hindlimb step quality, no statistically significant differences were observed between any groups (*p* > 0.05).

**Figure 17:**
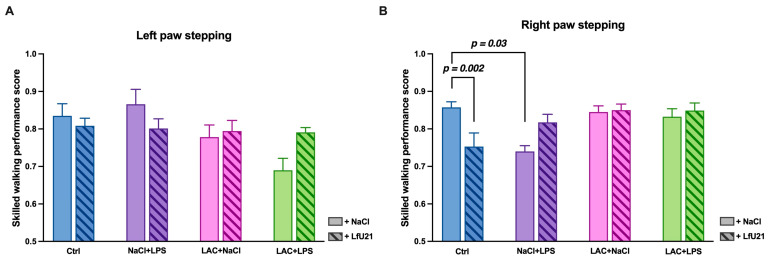
Effect of LfU21 on forelimb step quality in the Rung Ladder test. Step quality is shown for the left (**A**) and right (**B**) forelimbs across groups receiving lactacystin (LAC), lipopolysaccharide (LPS), or LAC + LPS, with or without LfU21 co-administration. Data are presented as mean ± SEM. Statistical analysis was performed using three-way ANOVA; significant differences are indicated on the graph.

When comparing the left forelimb step quality index, a trend toward decreased performance (*p* = 0.07) was observed in the LAC + LPS group compared to the control (Figure 17A).

Analysis of the right forelimb revealed more pronounced differences. Animals in the NaCl + LPS group showed significantly reduced step quality compared to controls (*p* = 0.03). Furthermore, control animals that received LfU21 (Ctrl + LfU21) exhibited a decrease in step quality relative to untreated controls (*p* = 0.002) (Figure 17B).

**Figure 18:**
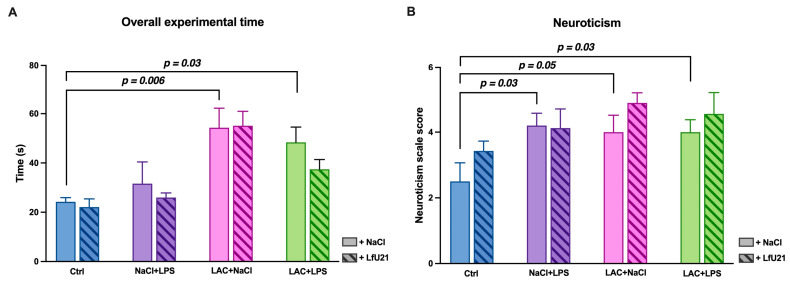
Effect of LfU21 on total test time and neuroticism score in the Rung Ladder test. (**A**) Total time to complete the test. (**B**) Neuroticism score. Data are presented for groups receiving lactacystin (LAC), lipopolysaccharide (LPS), or LAC + LPS, with or without LfU21 co-administration. Values are mean ± SEM. Statistical analysis was performed using three-way ANOVA; significant differences are indicated on the graph.

Analysis of the total test time showed that animals receiving LAC required significantly more time to complete the test compared to controls (*p* = 0.006). A similar increase in total time was observed in the group exposed to the combined LAC and LPS model (*p* = 0.03). LfU21 administration had no significant effect on this measure (*p* > 0.05) (Figure 18A).

For neurotization scores, an increase was noted in animals that received LPS or LAC alone, as well as in those exposed to the combined LAC+LPS model (*p* = 0.05, *p* = 0.03, and *p* = 0.03, respectively) (Figure 18B).

#### 2.6.2. Assessment of Motor Activity in the “Open Field” Test

**Figure 19:**
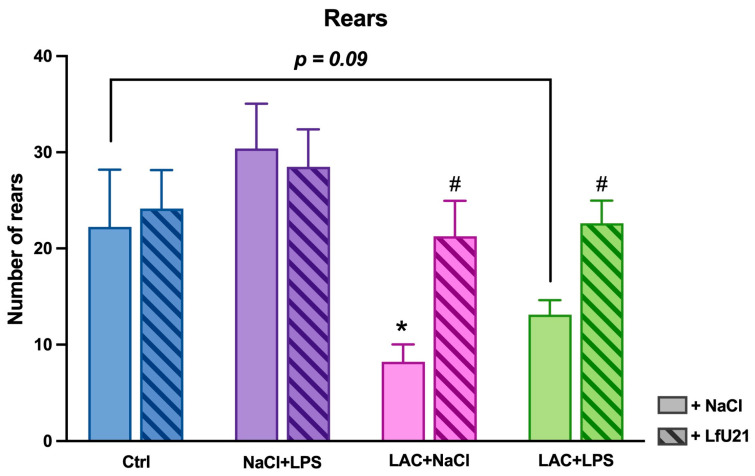
Effect of LfU21 on vertical motor activity in the Open Field test. The number of rears is shown for groups receiving lactacystin (LAC), lipopolysaccharide (LPS), or LAC + LPS, with or without LfU21 co-administration. Data are presented as mean ± SEM. Statistical analysis was performed using three-way ANOVA. Significance indicators: *—*p* < 0.05 vs. Ctrl; #—*p* < 0.05 vs. the corresponding group without LfU21.

In the Open Field test, a significant decrease in the number of rears was observed in the LAC group (*p* = 0.02) and a trend toward a decrease in the LAC + LPS group (*p* = 0.09). LfU21 administration prevented this reduction both in animals receiving LAC alone (*p* = 0.01) and in those with the combined PD model (*p* = 0.03) (Figure 19).

**Figure 20:**
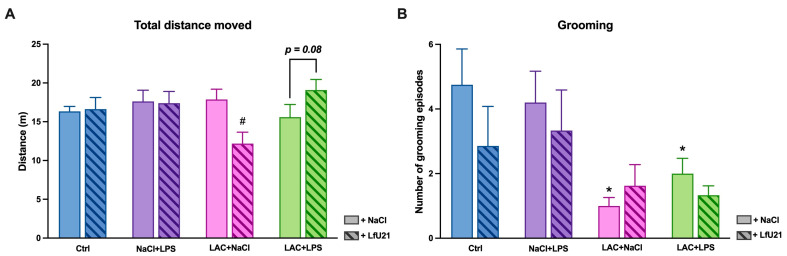
Effect of LfU21 on horizontal motor activity and grooming in the Open Field test. (**A**) Distance traveled. (**B**) Number of grooming episodes. Data are presented for groups receiving lactacystin (LAC), lipopolysaccharide (LPS), or LAC + LPS, with or without LfU21 co-administration. Values are mean ± SEM. Statistical analysis was performed using three-way ANOVA. Significance indicators: *—*p* < 0.05 vs. Ctrl; #—*p* < 0.05 vs. the corresponding group without LfU21.

The drug also exhibited a multidirectional effect on horizontal motor activity. Animals receiving LAC alone showed a significant decrease in distance traveled (*p* = 0.004), whereas those with the combined model displayed a trend toward increased distance (*p* = 0.08) (Figure 20A).

Furthermore, animals treated with LAC alone and those with the combined PD model exhibited a significant reduction in the number of grooming episodes compared to the control group (*p* = 0.007 and *p* = 0.03, respectively) (Figure 20B).

## 3. Discussion

Parkinson’s disease is characterized by functional impairments across numerous genes and multiple organ systems throughout its progression. This multifaceted pathophysiology motivated the selection of *Limosilactobacillus fermentum* U-21 (LfU21) as a candidate live biotherapeutic. Prior multi-omics studies have demonstrated that this strain synthesizes several pharmacologically active substances with potential anti-Parkinsonian properties [21,22,23]. The multifunctionality of these synthesized ingredients suggests that LfU21 may be capable of addressing several impaired biological pathways concurrently. A critical next step was to establish a suitable combined model of PD.

In this study, we developed a combined PD model designed to simulate the multifactorial etiology of PD and recapitulate its key pathological features. The first “hit” was the neurotoxic effect of intranigral lactacystin, a proteasome inhibitor. Its active metabolite, clasto-lactacystin β-lactone, irreversibly inhibits the 20S proteasome complex, promoting the pathological accumulation and aggregation of α-synuclein [27]. The second “hit” was systemic immune activation via intraperitoneal administration of lipopolysaccharide (LPS). This bacterial endotoxin acts as a pathogen-associated molecular pattern (PAMP), activating pattern recognition receptors (PRRs) and triggering a robust cytokine response. Peripheral cytokines can subsequently enter the central nervous system (CNS) via circumventricular organs or signal through vagal afferents, collectively promoting neuroinflammation. This process involves local cytokine production by activated microglia and astrocytes, ultimately contributing to dopaminergic neuron loss and exacerbating disease pathology [28].

In this study, we employed a multi-system panel of biomarkers to assess key pathological aspects of Parkinson’s disease (PD). The brain, liver, and intestine were selected as primary sites of analysis. In the brain, we measured tyrosine hydroxylase and IBA1 protein levels, along with the expression of the *drd2*, *ngf*, *bdnf*, and *trkB* genes. In the liver, we evaluated redox status by quantifying superoxide dismutase (SOD), catalase (CAT), and reduced glutathione (GSH), and assessed inflammation via levels of IL-6, IL-10, and TNF. In the intestine, we analyzed total and phosphorylated α-synuclein levels and counted goblet cells. In addition to these molecular biomarkers, behavioral outcomes were measured using the “Rung Ladder” and “Open Field” tests to evaluate motor function and overall condition.

Intranigral administration of lactacystin, either alone or combined with systemic lipopolysaccharide (LPS), induced significant degeneration of the dopaminergic system and microglial activation in the substantia nigra pars compacta. These findings align with prior studies demonstrating that the combined inhibition of the proteasome and induction of systemic inflammation exacerbates neurotoxicity and recapitulates core PD pathologies, including dopaminergic neuron loss and microgliosis [29]. Despite the documented antioxidant and neuroprotective potential of LfU21, its administration in this model did not mitigate these neurodegenerative or neuroinflammatory changes. This contrasts with previous work in which oral LfU21 attenuated the glial response and reduced phosphorylated α-synuclein accumulation in a paraquat-LPS model, although it similarly failed to prevent neuronal death [30].

The dopamine D2 receptor (DRD2), one of five dopamine receptor subtypes in humans, is highly expressed in the pituitary gland and central nervous system [31]. It regulates dopaminergic signaling, motor control, and cognitive processes. Mice with reduced DRD2 density exhibit a heightened neuroinflammatory response and increased vulnerability of dopaminergic neurons to MPTP-induced toxicity [32]. Conversely, elevated D2 receptor levels are observed in rats with significant dopamine deficits and in the putamen during early stages of sporadic Parkinson’s disease (PD) in humans [33]. Thus, *drd2* gene expression may serve as a biomarker for dopamine deficiency.

Consistent with reports of increased receptor activation in early PD, our study found elevated *drd2* expression in the right striatum of rats receiving LPS compared to control, combined PD model (LAC + LPS), and lactacystin-only groups. An LPS-related effect was also apparent in the left striatum, where *drd2* expression in the combined model group was higher than in the lactacystin-only group.

Nerve growth factor (NGF) is a neurotrophic factor essential for the development and survival of sympathetic, sensory, and cholinergic neurons [34]. Neurodegenerative diseases are frequently associated with impaired synthesis or release of neurotrophic factors, including NGF [35,36]. However, in our study, *ngf* gene expression remained unaltered in the right and left striatum across all experimental groups, suggesting that the factors tested (LPS and lactacystin) have minimal influence on its regulation.

Another key member of the neurotrophin family is brain-derived neurotrophic factor (BDNF). BDNF supports neuronal survival, maturation, synaptogenesis, and plasticity in the central nervous system [37]. In PD, BDNF levels are reduced in the nigrostriatal pathway, correlating with neurodegeneration and clinical decline [38]. BDNF exerts its effects primarily through the neuronal receptor tyrosine kinase-2 (TrkB), forming the BDNF/TrkB signaling pathway. This pathway is notably downregulated in PD, with its reduction positively correlating with disease severity and duration [39,40]. Consequently, augmenting BDNF/TrkB signaling is an active therapeutic strategy for mitigating PD pathology [41].

Thus, analyzing *bdnf* and *trkB* expression can indicate the extent of pathology and serve as a measure of model fidelity. In our study, *bdnf* expression was reduced in the combined PD model and in groups exposed to LPS or lactacystin alone, confirming that both individual and combined insults replicate this pathological feature. Administration of LfU21 prevented this pronounced decrease in *bdnf* expression.

Furthermore, a statistically significant decrease in *bdnf* expression was observed in the right striatum—the site of lactacystin injection—in the combined model and in groups treated with LPS or lactacystin alone. In the left striatum, a significant decrease occurred only in the LPS-only group, indicating distinct hemispheric effects of the administered substances. LfU21 also exhibited a divergent, hemisphere-specific influence: it significantly increased *bdnf* expression in the left striatum while decreasing it in the right. This suggests that LfU21 may engage different pharmacologically active ingredients (PAIs) and mechanisms in each hemisphere, highlighting an important avenue for future mechanistic research. Functional asymmetry of the human and animal brain is a rapidly developing field of science [42,43]. The visible difference between gene expression in the right and left striatum can be explained by the possible asymmetrical response of the left and right sides to PAIs and signals from controlled organs and systems. But it is unknown under whose control the intestines and abdominal cavity (where LPS is introduced) are. And the most global question is which part of the brain communicates with the gut microbiome in the famous gut–brain axis. We also noted a baseline hemispheric difference in control animals, with higher *bdnf* expression in the right striatum—a finding consistent with previous reports [44]. BDNF acts through TrkB receptor signaling, a pathway known to be impaired in PD [41]. In our study, *trkB* expression remained unchanged across all groups. This indicates that within our combined PD model, BDNF/TrkB signaling is likely constrained by reduced *bdnf* expression rather than by alterations in its receptor.

Reactive oxygen species (ROS) are established mediators of neurological dysfunction. LPS induces neuroinflammation and neurodegeneration through ROS generation and subsequent oxidative stress [45,46]. Key markers of this process include superoxide dismutase (SOD), catalase (CAT), and reduced glutathione (GSH) [47]. Oxidative stress typically manifests as elevated SOD and CAT activity alongside depleted GSH.

Glutathione, a critical cellular antioxidant, exists in reduced (GSH) and oxidized (GSSG) forms. The GSH/GSSG ratio is a key indicator of cellular redox balance, protecting cells by neutralizing free radicals and degrading peroxides. GSH also serves as a substrate for detoxifying enzymes, providing defense against toxins and inflammation [48]. During oxidative stress, GSH levels decline [49,50]. In our experiments, GSH was significantly reduced in the NaCl + LPS and LAC + LPS groups. LfU21 prevented this decrease in both the NaCl + LPS + LfU21 and LAC + LPS + LfU21 groups. The most pronounced protective effect was observed in the LAC + LPS + LfU21 group compared to its untreated counterpart. Notably, LfU21 elevated GSH above control levels, though it did not significantly alter GSH in the LAC + NaCl group, where no initial depletion occurred.

Superoxide dismutase (SOD) is crucial for maintaining the in vivo redox balance, catalyzing the dismutation of superoxide anions into oxygen and hydrogen peroxide to protect cells from oxidative damage. Typically, SOD activity rises during acute oxidative stress but can decline under prolonged conditions [51]. Consistent with an acute stress response, we observed elevated SOD levels in the NaCl + LPS and LAC + LPS groups. LfU21 prevented this increase in the corresponding treatment groups (NaCl + LPS + LfU21 and LAC + LPS + LfU21) without affecting SOD activity in controls.

Catalase (CAT), an iron-porphyrin enzyme, efficiently decomposes hydrogen peroxide, and its activity generally correlates with the degree of oxidative stress [52]. Our experiments confirmed increased CAT activity in the LAC + LPS and NaCl + LPS groups. LfU21 normalized CAT activity to control levels in the NaCl + LPS + LfU21 group, though this effect was less pronounced in the LAC + LPS + LfU21 group. LfU21 alone did not alter CAT activity in control animals. Overall, LfU21 significantly contributed to restoring normal levels of antioxidant enzymes and glutathione in groups exposed to LPS.

An immune response is central to PD pathogenesis, with dysregulation of innate and adaptive immunity driving central and peripheral inflammation. Cytokines are key modulators, coordinating both pro- and anti-inflammatory pathways [53]. In PD patients, upregulation of TNF and IL-6 is well-documented in cerebrospinal fluid, brain tissue, and peripheral blood [54,55,56], with blood IL-6 levels correlating with disease severity [57]. Similarly, serum levels of the anti-inflammatory cytokine IL-10 are elevated and correlate with gastrointestinal dysfunction [58]. Given the liver’s central role in systemic inflammation, we measured cytokine levels in hepatic tissue, which also offers greater sensitivity for ELISA detection [59]. In line with the literature [60,61], we found elevated levels of both pro-inflammatory (TNF, IL-6) and anti-inflammatory (IL-10) cytokines in LPS-treated rats. LfU21 prevented this increase, demonstrating significant immunomodulatory properties. Differences in IL-6 and IL-10 levels between the combined model and lactacystin-only groups further underscore the pronounced impact of LPS. Interestingly, LPS alone induced stronger TNF activation than in combination with lactacystin, a counterintuitive antisynergism warranting further investigation.

Intestinal dysbiosis and the proliferation of toxin- and LPS-producing bacteria are considered potential triggers for PD [8]. This can compromise the intestinal epithelial barrier, elevating systemic LPS levels [62]. Local exposure to LPS also drives intestinal inflammation, which in turn upregulates α-synuclein expression within the enteric nervous system [62].

A key morphological indicator of intestinal inflammation is an increase in goblet cell numbers and mucus production. In our study, goblet cell counts in the intestinal villi epithelium were elevated in the LPS, LAC, and LPS + LAC treatment groups. Goblet cell-derived mucus forms a protective barrier against pathogen invasion [63]. The observed increase likely reflects the epithelial stress response to systemic inflammation (from LPS) and central nigrostriatal damage (from lactacystin). Indeed, neurodegeneration in the nigrostriatal system can induce intestinal inflammatory changes, including goblet cell hyperplasia [64]. In our model, this increase was accompanied by elevated levels of total and phosphorylated α-synuclein in the myenteric plexus ganglia. Interestingly, contrary to expectations, combining LPS with lactacystin did not exacerbate α-synuclein phosphorylation in the intestine.

Given the well-established link between gut microbiota and neurodegenerative diseases like PD, modulating the microbiome represents a promising therapeutic avenue [65,66,67,68]. According to the “gut–brain axis” hypothesis, early α-synuclein pathology originates in enteric nervous tissue [69], potentially initiating the cascade of central neurodegeneration. Thus, interventions that reduce enteric α-synuclein accumulation could slow or halt disease progression. We investigated the effect of LfU21 on goblet cell numbers and α-synuclein accumulation in rats exposed to LPS, LAC, or their combination. LfU21 administration prevented the LPS-induced increase in goblet cells, suggesting an anti-inflammatory effect in the intestine. It also effectively reduced α-synuclein accumulation in the myenteric plexus, supporting its therapeutic potential for early-stage PD.

In our study, combined LPS and LAC exposure impaired motor performance in the Rung Ladder test, reducing step quality and increasing completion time. These results align with prior work by Deneyer et al. [29], who demonstrated similar motor deficits in a comparable model using the Rotarod test. Furthermore, motor impairments—whether from combined or individual toxin exposure—were accompanied by non-motor symptoms, reflected in heightened neurosis-like behavior as measured by a standardized scale. This finding is consistent with other reports, such as anxiety-like behavior in rats following 6-OHDA administration [70].

The Open Field test revealed that lactacystin administration had the most pronounced effect on motor symptoms. Animals receiving lactacystin alone exhibited reduced vertical motor activity (rearing) and fewer grooming episodes, likely due to impaired nigrostriatal signaling, which is essential for coordinated motor patterns [71].

The influence of LfU21 on motor performance was bidirectional. While it prevented the decline in step quality in animals with the PD model, it paradoxically worsened performance in healthy controls. This suggests the drug’s effect is context-dependent: under pathological conditions, it may confer protection by modulating inflammatory processes during neurodegeneration [72,73,74], in a healthy state, it might induce a low-grade systemic inflammatory response [75]. Therefore, the modulatory action of LfU21 appears most relevant in disease states and is influenced by the host’s physiological baseline.

These findings align with previous studies showing that probiotics and prebiotics (e.g., *Lactobacillus salivarius* AP-32, *Bifidobacterium animalis* ssp. *lactis* Bb12, *Lactobacillus rhamnosus* GG) can alleviate motor deficits in various rodent PD models in tests such as the “rotarod”, “apomorphine test”, “cylinder”, and “narrowing beam” [76,77].

Notably, LfU21 did not significantly affect the time required to complete the Rung Ladder test. This indicates that its protective effect is primarily on movement quality (e.g., step coordination) rather than on overall speed.

In summary, our results demonstrate that LfU21 exerts a protective effect on motor function in a combined LAC + LPS model of PD, likely mediated by its antioxidant properties and ability to reduce intestinal α-synuclein accumulation.

A summary of the biomarker changes observed across the combined PD model, its individual components, and their modulation by LfU21 is presented in Table 1.

Table S1 lists genes and proteins of the *Limosilactobacillus fermentum* U-21 strain identified as potential determinants of its pharmacological activity against Parkinson’s disease (PD). The bioactive substances encoded by these genes may act through several pathways: (1) interaction with Toll-like receptors (TLRs) on intestinal epithelial cells [78]; (2) direct entry into the bloodstream [79,80,81]; or (3) vesicular packaging and transport [82]. LfU21 produces several amino acids—including histidine, glycine, and arginine—which can influence central nervous system processes through intestinal metabolism [83,84,85]. The strain also secretes the ATP-dependent Clp protease, which exhibits protein disaggregase activity, along with other proteases (e.g., zinc metalloprotease HtpX and ATP-dependent zinc metalloprotease FtsH) that may contribute to reducing intestinal α-synuclein levels [86]. Additionally, LfU21 produces components of the thioredoxin complex, enhancing its antioxidant capacity [87].

Elucidating and refining the mechanisms of these PAIs for the targeted correction of specific PD biomarkers remains a crucial yet challenging step in advancing LfU21 as a live biotherapeutic product (LBP) for PD.

In Parkinson’s disease, a fundamental limitation for treatment is the inability to diagnose the early stages of its manifestation and the trigger mechanisms of its onset. It is assumed that one of these mechanisms is triggered in the intestine and is caused by negative factors arising from an imbalance in the composition of the microbiota. For this reason, the search for and study of biomarkers of disease triggers in the intestine and the enteric and central nervous systems is crucial. Besides those studied in this work, additional biomarkers that may change in the context of PD development include malondialdehyde, which level increases with elevated oxidative stress, DJ-1 protein (associated with oxidative stress and neuroprotection), NfL (neurofilament light chain, neuronal protein released during axonal damage), tau proteins, glial marker GFAP, miRNAs, and others [88,89,90,91]. Epigenetic mechanisms appear to play an important role in the onset of Parkinson’s disease and at all stages of its development [92]. Today, intensive research is being conducted on human and rodent biomaterials on the involvement of DNA methylation in the expression of putative biomarkers of Parkinson’s disease [93,94,95]. At the same time, there are virtually no studies on the influence of functional bacteria of the microbiome on these processes. It seems necessary and is planned to investigate the role of LfU21 in epigenetic mechanisms, primarily in DNA methylation and its influence on the expression of certain neurotrophic genes and the alpha-synuclein gene. It also seems necessary to study changes in the composition of the microbiota in a combined model of PD and LfU21 administration, which could help identify the mechanism of action of LfU21 in the context of microbiome modulation.

## 4. Materials and Methods

### 4.1. Bacterial Strain

The strain *Limosilactobacillus fermentum* U-21 (NCBI Reference Sequence: NZ_CP103293.1), held in the collection of the Laboratory of Bacterial Genetics at the Vavilov Institute of General Genetics, Russian Academy of Sciences, was isolated from an astronaut’s fecal sample. It is deposited in the All-Russian Collection of Industrial Microorganisms under accession number B-12075.

### 4.2. Culture Media, Growth Conditions and Lyophilization

*Limosilactobacillus fermentum* U-21 was cultivated in a proprietary industrial-type culture medium under controlled fermentation. The production medium consisted of: 75% whey (from 12% skim milk powder), 7% lactopeptone, 2% yeast extract, 2% inulin, 0.002% ascorbic acid, 0.05% sodium chloride, 0.005% manganese sulfate, and 0.02% magnesium sulfate. Cultivation was performed in laboratory fermenters (Prointech, Pushchino, Russia) at 37 °C and 100 rpm without aeration, with an initial medium pH of 6.8.

The production medium was inoculated with 2% (*v*/*v*) of a seed culture. The seed material was prepared by first culturing the strain on MRS agar (HiMedia, Mumbai, India) and then in MRS broth. The MRS broth contained (per liter): proteose peptone, 10 g; beef extract, 10 g; yeast extract, 5 g; dextrose, 20 g; Tween-80, 1 g; ammonium citrate, 2 g; sodium acetate, 5 g; magnesium sulfate, 0.1 g; manganese sulfate, 0.05 g; and disodium hydrogen phosphate, 2 g. The solid MRS agar medium had an identical composition, except disodium hydrogen phosphate was replaced with dipotassium hydrogen phosphate (2 g/L), and agar-agar (12 g/L) was added.

For lyophilization, a 20-h culture (3 × 10^9^ CFU/mL) was centrifuged at 7000× *g* and 4 °C for 10 min. The pellet was washed with phosphate-buffered saline (PBS: 1.7 mM KH_2_PO_4_, 5.2 mM Na_2_HPO_4_, 150 mM NaCl, pH 7.4) and resuspended in a lyoprotectant solution (10% sucrose, 1% gelatin). The suspension was held at −20 °C for 24 h and then freeze-dried at −52 °C and 0.42 mbar for 72 h using a Labconco 2.5 lyophilizer (Labconco, Kansas City, MO, USA). The resulting lyophilisates were stored at 4 °C. Viability and titer were verified prior to experimental use.

### 4.3. Animal Collection and Maintenance

All procedures were performed in compliance with ethical standards for the care and use of laboratory animals and were approved by the Ethics Committee of the Russian Center of Neurology and Neurosciences (Protocol No. 2–4/25, dated 17 February 2025). Efforts were made to minimize the number of animals used.

The study utilized male Wistar rats (*n* = 82), aged 3.5 months, with initial body weights of 300–350 g. All animal handling and husbandry were conducted in accordance with Recommendation No. 33 of the Board of the Eurasian Economic Commission (14 November 2023, “Guidelines for Working with Laboratory (Experimental) Animals in Preclinical (Non-Clinical) Studies”) and the “Rules for Working with Laboratory Rodents and Rabbits” (GOST 33216-2014).

Animals were housed under standard vivarium conditions with a 12-h light/dark cycle, ad libitum access to food and water, and a 14-day acclimatization period prior to the start of the experiment.

### 4.4. Surgical Operations and Experimental Design

For stereotactic surgery, animals were secured in a stereotaxic frame (RWD, PRC). Following a scalp incision, burr holes were drilled according to coordinates from the Paxinos rat brain atlas [96]. A cotton-gauze pad was placed beneath each animal to prevent hypothermia during the procedure.

Lactacystin (LAC; ENZO, Basel, Switzerland) was dissolved in saline (4 µg in 3 µL) and injected unilaterally into the right substantia nigra pars compacta (coordinates: AP = −4.8 mm, L = 2.2 mm, V = 8.0 mm) of 43 animals. The contralateral side received an equal volume of saline. Sham-operated controls (*n* = 39) received bilateral saline injections. Three days post-surgery, a subset of animals began receiving intraperitoneal (i.p.) injections of E. coli O111:B4 lipopolysaccharide (LPS; Sigma-Aldrich, St. Louis, MO, USA) at 1 mg/kg, administered twice weekly for four weeks (total of 8 injections, 0.25 mL each). Two days post-surgery, oral administration of the pharmacobiotic *L. fermentum* U-21 (LfU21) commenced. The strain was suspended in saline at a concentration of 1.5 × 10^10^ CFU per 3 mL and administered daily at 0.3 mL (per os) [24].

Animals were allocated into eight experimental groups:

1. Ctrl (*n* = 9): Sham surgery + i.p. saline + oral saline.
2. Ctrl + LfU21 (*n* = 9): Sham surgery + i.p. saline + oral LfU21.
3. LAC + NaCl + NaCl (*n* = 11): LAC + i.p. saline + oral saline.
4. LAC + NaCl + LfU21 (*n* = 10): LAC + i.p. saline + oral LfU21.
5. NaCl + LPS + NaCl (*n* = 11): Sham surgery + i.p. LPS + oral saline.
6. NaCl + LPS + LfU21 (*n* = 10): Sham surgery + i.p. LPS + oral LfU21.
7. LAC + LPS + NaCl (*n* = 11): LAC + i.p. LPS + oral saline.
8. LAC + LPS + LfU21 (*n* = 11): LAC + i.p. LPS + oral LfU21.

**Figure 21:**
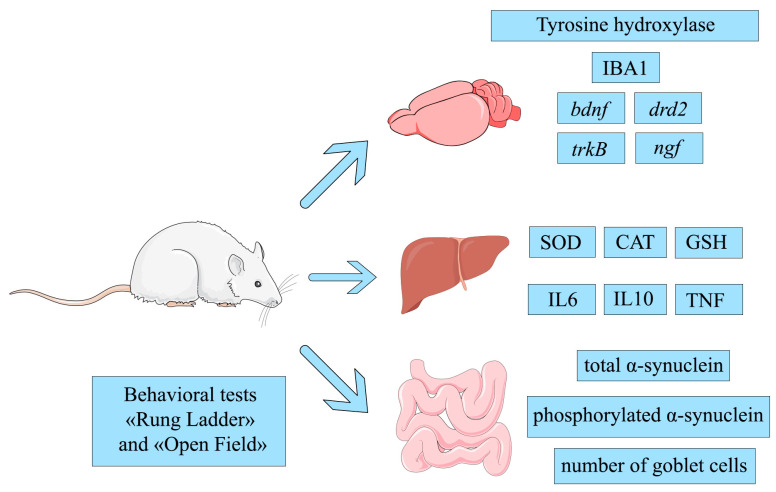
Overview of the measured biomarkers.

All animals were euthanized two months post-surgery for endpoint analysis (Figure 21).

### 4.5. Analysis of Behavioral Activity

To evaluate the neurotoxic effects of lactacystin and LPS, animal behavior was assessed eight weeks post-surgery using two standardized tests: the Rung Ladder test [97] and the Open Field test [98].

Rung Ladder Test: This test evaluates motor function and coordination by quantifying correct paw placements, slips, and misses. Performance was scored using the Skilled Walking Performance Score (SWPS) [99], and the total time to complete the test was recorded [100]. An advanced version of the apparatus, featuring irregularly spaced rungs (Open-Science, Moscow, Russia), was used to further assess forelimb–hindlimb coordination. The test also included an assessment of neuroticism.

Open Field Test: Motor activity and exploratory behavior were assessed in a PVC arena (78 × 78 × 40 cm) during a 3-min session.

All behavioral sessions were video-recorded and analyzed using the Any-Maze tracking system (Stoelting Inc., Wood Dale, IL, USA).

### 4.6. Transcription Analysis

#### 4.6.1. RNA Extraction and Reverse Transcription Reaction

Following the experimental endpoint, three animals per group were selected. Rats were euthanized by guillotine decapitation, and striatal samples were immediately collected on ice into IntactRNA solution (Eurogen, Moscow, Russia) and stored per the manufacturer’s instructions.

Total RNA was extracted from striatal tissue using ExtractRNA reagent (Eurogen, Moscow, Russia) according to the manufacturer’s protocol. The RNA pellet was resuspended in nuclease-free water (Eurogen, Moscow, Russia). RNA integrity was assessed by gel electrophoresis.

Subsequently, residual genomic DNA was removed by treating the samples with DNase I (diaGene, Moscow, Russia) as specified by the manufacturer. Complementary DNA (cDNA) was synthesized from the purified RNA using the MMLV Reverse Transcriptase kit (Eurogen, Moscow, Russia) according to the recommended protocol.

#### 4.6.2. Real-Time qPCR

The quantitative PCR (qPCR) reaction mixture was prepared using the qPCRmix-HS SYBR kit (Eurogen, Moscow, Russia) according to the manufacturer’s instructions. Reactions were performed on a CFX96 system (Bio-Rad, Hercules, CA, USA) with the following amplification program: initial denaturation at 95 °C for 5 min; followed by 40 cycles of denaturation at 95 °C for 30 s, annealing for 30 s at 57.6 °C (*drd2*), 64 °C (*ngf*), 54.7 °C (*bdnf*), or 62 °C (*trkB*), and extension at 72 °C for 30 s. A melting curve analysis was performed following amplification. Data were analyzed using CFX Manager software v3.1 (Bio-Rad, USA). Relative gene expression was calculated using the ∆∆Cq method with three biological replicates (individual animals) per group. The *actb* gene served as the endogenous reference [101]. Primers were designed using Primer-BLAST ver. 2.17.0 [102] (sequences are provided in Table A1).

### 4.7. Markers of Redox Potential Analysis

Rat liver samples were homogenized in liquid nitrogen and suspended in ice-cold phosphate-buffered saline (PBS; 0.01 M, pH 7.4) at a 1:10 (*w*/*v*) ratio. The homogenate was vortexed and centrifuged at 10,000× *g* for 10 min at 4 °C to remove insoluble debris. The resulting supernatant was used undiluted for glutathione (GSH) quantification and diluted 10-fold for catalase (CAT) and superoxide dismutase (SOD) activity assays.

Enzyme activities and GSH content were determined by colorimetric assay using the following commercial kits according to the manufacturers’ protocols: SOD (E-BC-K031-S, Elabscience, Wuhan, China), CAT (IS104/A001-1-2, Cloud-Clone Corp., Wuhan, China), and GSH (E-BC-K030-S, Elabscience, Wuhan, China).

### 4.8. The Study of the Immunomodulatory Activity of the Strain Using ELISA

Rat liver samples were homogenized on ice in freshly prepared lysis buffer (25 mM Tris-HCl, pH 7.4; 150 mM NaCl; 1 mM EDTA; 1% NP-40) using a glass homogenizer. The homogenate was sonicated on ice using a Vibra-Cell device (Sonics, Oklahoma, OK, USA) with the following parameters: 40% amplitude, 5 s pulse on, 10 s pulse off, for 15 cycles. The lysate was then centrifuged at 10,000× *g* for 15 min at 4 °C.

The supernatant was collected, and total protein concentration was quantified using a Qubit fluorometer (Invitrogen, Carlsbad, CA, USA). Cytokine levels (IL-6, IL-10, and TNF) were measured in the supernatants using commercial ELISA kits (Cloud-Clone Corp., Wuhan, China; catalog numbers: HEA133Ra, HEA079Ra, HEA056Ra) according to the manufacturer’s instructions. Absorbance was read at 450 nm on a DTX 880 Multimode Detector (Beckman Coulter, Brea, CA, USA).

### 4.9. Histological Preparations and Analysis

#### 4.9.1. Morphometric Study of the Substantia Nigra Pars Compacta

For morphological analysis, animals were euthanized by guillotine decapitation. Brains were removed, fixed in 10% neutral-buffered formalin for 24 h, and cryosectioned at 10 μm thickness using a Tissue-Tek Cryo3 Flex cryostat (Sakura FineTek, Torrance, CA, USA). Antigen retrieval was performed by heat treatment in citrate buffer (pH 6.0) for 15 min. Sections were incubated with primary antibodies in a humid chamber 18 h at room temperature.

To detect tyrosine hydroxylase, monoclonal rabbit antibodies (1:250, ab137869, Abcam, Cambridge, UK) and secondary polyclonal antibodies to rabbit immunoglobulins (1:250, goat, Anti-Rb, CF488, SAB4600045-250UL (Sigma, St. Louis, MO, USA)). To detect microglia, monoclonal rabbit antibodies to the IBA1 (ionized calcium-binding adapter molecule 1) protein (1:300, ab178847, Anti-IBA1 Rb (Abcam, Cambridge, UK)) and secondary polyclonal antibodies to rabbit immunoglobulins (1:250, goat, Anti-Rb, CF488, SAB4600045-250UL (Sigma, St. Louis, MO, USA)). The sections were counterstained with DAPI. For the study, 5–6 consecutive sections (selected approximately every 50 μm) of substantia nigra were selected in the area of intranigral lactacystin administration. For quantitative assessment, the region of interest (the compact and reticular parts of the substantia nigra on the left and right) was manually selected in ImageJ ver. 1.54p (NIH) from images obtained with the same microscope lighting settings (Nikon SMZ-18 (Nikon Instruments Inc., Tokyo, Japan)). The staining intensity of IBA1 was evaluated in the selected area. Similarly, by selecting the area of interest and using the Bernsen threshold segmentation algorithm, the areas stained for tyrosine hydroxylase (neuron bodies and their processes) were segmented in the substantia nigra, and the area fraction of immunostained tissue was evaluated.

#### 4.9.2. Morphometric Study of the Small Intestine

##### Immunofluorescence Staining for Total and Phosphorylated α-Synuclein in the Ganglia of the Myenteric Plexus of the Small Intestine of Wistar Rats

For immunomorphometric analysis, six animals were selected from each group. Sections of the small intestine approximately 10 cm long were excised at a distance of 30–40 cm from the pylorus and fixed in formalin for 24 h. They were then cut crosswise into 3 mm thick pieces, impregnated with a 30% sucrose solution, then with Tissue-Tek O.C.T. (“Optimal cutting temperature”) Compound (Sakura Finetek, Tokyo, Japan) cryoblock reagent, and cut on a cryostat into 10 μm thick sections. Immunofluorescence reactions were performed using an indirect method, with primary antibodies to total α-synuclein (Rb, Sigma (St. Louis, MO, USA), 1:300), α-synuclein phosphorylated at serine-129 (Ms, Abcam (Cambridge, UK), 1:250) and secondary antibodies (goat anti-mouse CF488, Sigma, 1:250 and goat anti-rabbit Alexa fluor 594, Abcam, 1:250). The preparations were examined and photographed under a Nikon Eclipse NiU microscope with a Nikon DS-Qi digital camera at 40× magnification with the same microscope lighting system settings. Morphometry was performed using ImageJ software ver. 1.54p (NIH) on photographs, with at least 30 fields of view per animal examined. The content of total and phosphorylated α-synuclein in the myenteric (intermuscular) ganglia was assessed indirectly by measuring the average intensity of fluorescent staining for these markers in the myenteric ganglia, the area of which was manually selected in the photographs, with correction for background staining.

##### Goblet Cells Count

Cryosections of small intestine were stained with Alcian blue and counterstained with hematoxylin. Sections were imaged at 25× magnification, and the number of goblet cells per 100 μm of villus length was quantified from the micrographs.

### 4.10. Statistical Analysis

Statistical analysis was performed using GraphPad Prism software (version 10.3.1; GraphPad Software, San Diego, CA, USA). A three-way analysis of variance (ANOVA) was applied to determine overall significance, followed by Fisher’s LSD post hoc test for pairwise group comparisons. Data are presented as mean ± standard error of the mean (SEM). Statistical significance was set at *p* < 0.05.

## 5. Conclusions

In this study, we identified key biomarkers modifiable by the live biotherapeutic drug LfU21 in a combined lactacystin (LAC) and lipopolysaccharide (LPS) rat model of Parkinson’s disease (PD). Currently, at least two primary mechanisms are proposed for how live biotherapeutic products (LBPs) exert their effects [8,76,103]. The first involves the direct action of pharmacologically active ingredients (PAIs) synthesized by the bacteria on specific host targets, delivered via the bloodstream or bacterial vesicles [103]. The second entails indirect modulation through beneficial changes in the gut microbiome composition. While this study did not elucidate the precise delivery mechanisms of PAIs to target organs—a necessary future research direction for LfU21 and other LBPs—it underscores a central challenge: identifying the specific PAIs responsible for therapeutic effects in PD and delineating their molecular mechanisms of action. LfU21 represents a pioneering candidate in the development of LBPs for PD. Looking forward, a strategic approach will involve using omics technologies and comprehensive databases to select synergistic combinations of LBPs derived from commensal bacteria. Given the proposed role of gut-initiated inflammation in PD pathogenesis, a priority is to identify LBPs that restore intestinal barrier integrity. This effort should be guided by a functional architecture model of PD [8], integrating host and microbial biomarker networks. Artificial intelligence, including machine learning, could be instrumental in this systems-level analysis. More focused tasks also remain, such as conducting metabolomic analyses to discover functionally significant biomarkers in feces, serum, and other accessible tissues. Ultimately, translating these preclinical findings into well-defined patient cohorts is a critical next step for clinical development.

## Figures and Tables

**Table 1:** Effect of lipopolysaccharide (LPS) and lactacystin (LAC), alone and in combination, on the studied biomarkers in rats. Arrows indicate a decrease (↓) or increase (↑) in each measurement relative to the control group. r, right striatum; l, left striatum.

Experimental Group	Without *L. fermentum* U-21	+ *L. fermentum* U-21	Indicators Normalized by *L. fermentum* U-21
Ctrl		*bdnf*(l) ↑, *bdnf*(r) ↓, total α-synuclein ↓, GSH ↑, upward forepaw stepping ↓ in the “Rung Ladder” test, quality of stepping with right limbs in the “Rung Ladder” test ↓	
NaCl + LPS	*drd2*(r) ↑, *bdnf*(l) ↓, *bdnf*(r) ↓, total α-synuclein ↑, phosphorylated α-synuclein ↑, number of goblet cells ↑, SOD ↑, CAT ↑, GSH ↓, IL6 ↑, IL10 ↑, TNF ↑, quality of right limb stepping in the “Rung Ladder” test ↓, level of neuroticism in the “Rung Ladder” test ↑	*bdnf*(r) ↓, total α-synuclein ↑, phosphorylated α-synuclein ↑,	*drd2*(r), *bdnf*(l), number of goblet cells, SOD, CAT, GSH, IL6, IL10, TNF
LAC + NaCl	Tyrosine hydroxylase (r) ↓, IBA1 ↑, *bdnf*(r) ↓, total α-synuclein ↑, phosphorylated α-synuclein ↑, number of goblet cells ↑, SOD ↓, testing time in the “Rung Ladder” test ↑, neuroticism level in the “Rung Ladder” test ↑, vertical motor activity in the “Open Field” test ↓, number of grooming acts in the “Open Field” test ↓	Tyrosine hydroxylase (r) ↓, IBA1 ↑, *bdnf*(r) ↓, phosphorylated α-synuclein ↑	total α-synuclein, SOD, vertical motor activity in the “Open Field” test
LAC + LPS	Tyrosine hydroxylase (r) ↓, IBA1 ↑, *bdnf*(r) ↓, total α-synuclein↑, phosphorylated α-synuclein↑, number of goblet cells ↑, SOD ↑, CAT ↑, GSH ↓, upward forepaw stepping ↓ in the “Rung Ladder” test, testing time in the “Rung Ladder” test ↑, neuroticism level in the “Rung ladder” test ↑, number of grooming acts in the “Open field” test ↓	Tyrosine hydroxylase (r) ↓, IBA1 ↑, *bdnf*(r) ↓, phosphorylated α-synuclein ↑, SOD ↓	total α-synuclein, GSH, upward forepaw stepping in the “Rung Ladder” test

## Data Availability

The original contributions presented in this study are included in the article/Supplementary Material. Further inquiries can be directed to the corresponding author.

## Supplementary Materials

The following supporting information can be downloaded at https://www.mdpi.com/article/10.3390/ijms27010446/s1.

## Abbreviations

The following abbreviations are used in this manuscript:

PD	Parkinson’s disease
LfU21	*Limosilactobacillus fermentum* U-21
LPS	lipopolysaccharide
LAC	lactacystin
ENS	enteric nervous system
CNS	central nervous system
PNS	peripheral nervous system
PAI	pharmacologically active ingredients
SOD	superoxide dismutase
CAT	catalase
GSH	reduced glutathione
BDNF	brain-derived neurotrophic factor
TrkB	neuronal receptor tyrosine kinase-2
NGF	nerve growth factor
DRD2	D2 dopamine receptor
IL6	Interleukin 6
IL10	Interleukin 10
TNF	tumor necrosis factor
ELISA	enzyme-linked immunosorbent assay

## Appendix A

**Table A1:** Primers used for qPCR.

Target Gene	R/F ^1^	Nucleotide Sequence (5′ → 3′)	NCBI RefSeq
*ngf*	F	GTTTTGCCAAGGACGCAGCTTTC	NM_001277055.1
	R	GTTCTGCCTGTACGCCGATCAA	
*drd2*	F	GGCCCTTCAATGGGTCAGAA	NM_012547.3
	R	GTAGACCACAAAGGCAGGGT	
*bdnf*	F	GAGCGTGTGTGACAGTATTAG	NM_001270635.1
	R	GTAGTTCGGCATTGCGAGTTC	
*trkB*	F	GTGGAGGAAGGGAAGTCTGTG	XM_008771425.4
	R	CAGTGGTGGTCTGAGGTTGGA	
*βactin*	F	AAGGCCAACCGTGAAAAGAT	NM_031144.3
	R	TGGTACGACCAGAGGCATAC	

^1^ F—forward sequence, R—reverse sequence.
