# Supplementary material for: Modulation of Behavioral, Biochemical, Immunomodulatory, and Transcriptional Profiles by the Strain Limosilactobacillus fermentum U-21 in Combined Model of Parkinson’s Disease in Wistar Rats
Source: Int J Mol Sci. 2025 Dec 31;27(1):446. doi: 10.3390/ijms27010446 (PMC12786881; doi:10.3390/ijms27010446)
Supplement: Supplementary file 1 [file ijms-27-00446-s001.zip › ijms-4042373-supplementary.pdf]

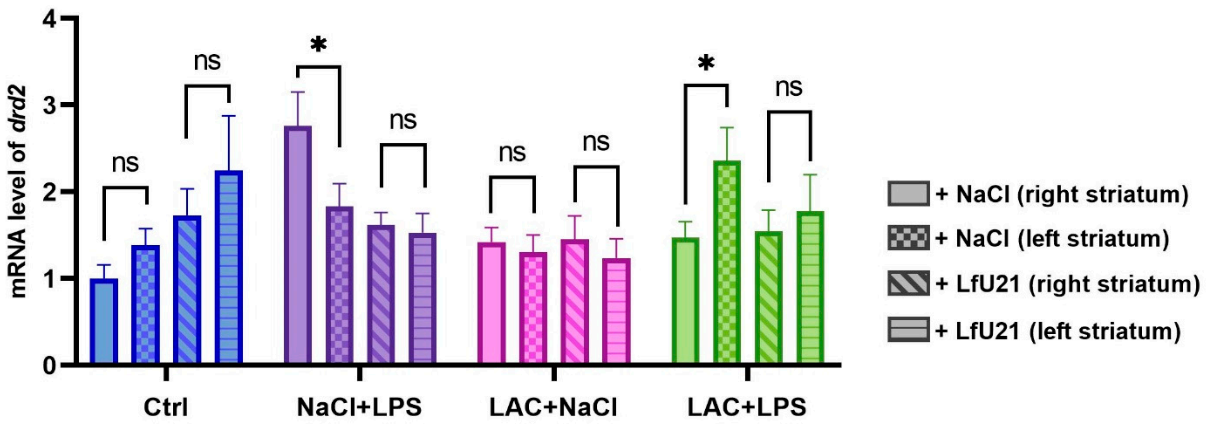

Figure S1. Expression of *drd2* mRNA in the right and left striatum. Gene expression levels were normalized to *actb* cDNA. Data are presented as mean ± SEM from three biological replicates, each measured in triplicate. Expression in the right striatum in the untreated control (Ctrl) group was set to 1. Statistical analysis was performed using three-way ANOVA. Significance indicators: ns – p>0.05, * - p < 0.05.

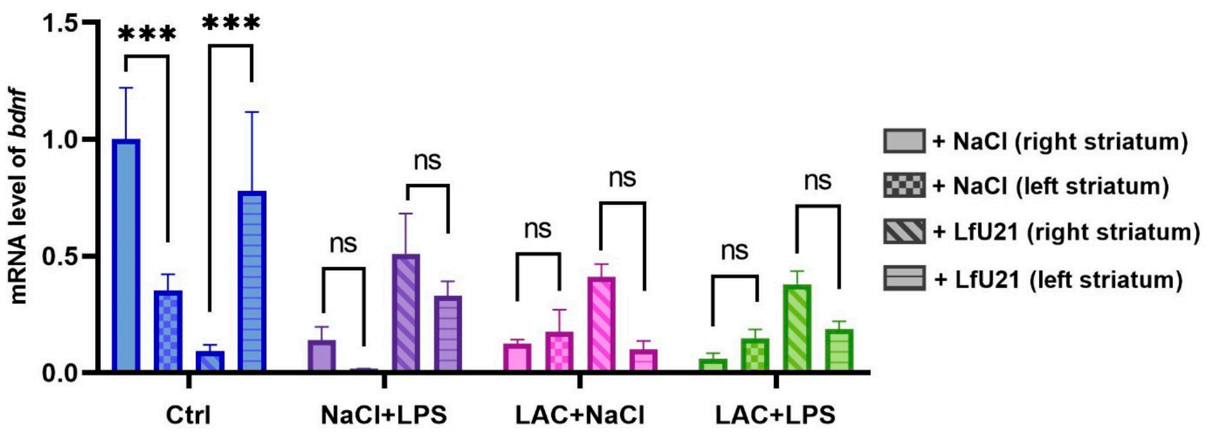

Figure S2. Expression of *bdnf* mRNA in the right and left striatum. Gene expression levels were normalized to *actb* cDNA. Data are presented as mean ± SEM from three biological replicates, each measured in triplicate. Expression in the right striatum in the untreated control (Ctrl) group was set to 1. Statistical analysis was performed using three-way ANOVA. Significance indicators: ns – p>0.05, *** - p < 0.001.

Table S1. Characterization of the gene (protein) composition of the *Limosilactobacillus fermentum* U-21 strain with potential anti-parkinsonian properties. Asterisks (*) indicate the presence of these products in the vesicles.

Name of the protein	Gene ID (Locus tag)	Protein ID	Protein function
**Factors of intestinal colonization, aggregation, and adhesion**			
LysM peptidoglycan-binding domain-containing protein*	C0965_005050	UVZ03164.1	Proteins containing a domain that binds to peptidoglycans
	C0965_007730	UVZ01776.1	
	C0965_010570	UVZ02258.1	
	C0965_002490	UVZ02712.1	
	C0965_003180	UVZ02840.1	

class A sortase*	C0965_001300	UVZ02498.1	Transpeptidase, which is involved in the attachment of surface proteins to the cell wall
**Interaction with the host organism**			
Zinc-dependent alcohol dehydrogenase*	C0965_001115	UVZ02466.1	Alcohol dehydrogenase activity; serotonin metabolism
Bifunctional acetaldehyde-CoA/alcohol dehydr ogenase*	C0965_001825	UVZ02586.1	
NAD(P)-dependent alcohol dehydrogenase	C0965_005655	UVZ01402.1	
NAD(P)-dependent alcohol dehydrogenase*	C0965_007735	UVZ01777.1	
Alcohol dehydrogenase AdhP*	C0965_008855	UVZ01965.1	
Zinc-dependent alcohol dehydrogenase family*	C0965_009215	UVZ03284.1	
**Synthesis of vitamins and amino acids**			
GTP cyclohydrolase I FolE*	C0965_007570	UVZ01744.1	Synthesis of folate
6,7-dimethyl-8-ribityllumazine synthase*	ribH - C0965_004500	UVZ03059.1	Synthesis of riboflavin
Bifunctional 3,4-dihydroxy-2-butanone-4-phosphate synthase/GTP cyclohydrolase II	C0965_004505	UVZ03060.1	
Riboflavin synthase	C0965_004510	UVZ03061.1	
Bifunctional diaminohydroxyphosphoribosyl aminopyrimidine d eaminase/5-amino-6-(5-phosphoribosylamino)uracil reductase*	ribD C0965_004515	UVZ03062.1	
Histidinol-phosphate transaminase*	C0965_004440	UVZ01744.1	Synthesis of histidine
Serine hydroxymethyltransferase*	C0965_002710	UVZ02749.1	Conversion of serine to glycine
Argininosuccinate lyase*	C0965_009490	UVZ02070.1	Synthesis of arginine
Argininosuccinate synthase*	C0965_009495	UVZ02071.1	

Ornithine carbamoyltransferase*	C0965_001935	UVZ02607.1	
Asparagine synthase B*	C0965_003855	UVZ02946.1	Synthesis of asparagine
Aspartate--ammonia ligase*	C0965_009340	UVZ02047.1	
Asparagine synthase (glutamine-hydrolyzing)*	C0965_010655	UVZ02273.1	
Aspartate aminotransferase family protein*	C0965_009460	UVZ02068.1	Conversion of L-aspartate to L-glutamate; carbon and nitrogen metabolism
PLP-dependent aspartate aminotransferase family protein*	C0965_005660	UVZ01403.1	
PLP-dependent aspartate aminotransferase family protein*	C0965_004700	UVZ03097.1	
PLP-dependent aspartate aminotransferase family protein*	C0965_004695	UVZ03096.1	
**Chaperones**			
Zinc metalloprotease HtpX*	C0965_001325	UVZ02503.1	
Hsp20/alpha crystallin family protein*	C0965_007720	UVZ01774.1	
Nucleotide exchange factor GrpE*	C0965_004405	UVZ03044.1	
Molecular chaperone DnaK*	C0965_004410	UVZ03045.1	
Co-chaperoneGroES*	C0965_002075	UVZ02634.1	
Chaperonin GroEL*	C0965_002080	UVZ02635.1	
ATP-dependent zinc metalloprotease FtsH*	C0965_001395	UVZ02517.1	
Molecular chaperone DnaJ*	C0965_004415	UVZ03046.1	
Hsp33 family molecular chaperone HslO*	C0965_001400	UVZ02518.1	
ATP-dependent Clp protease proteolytic subunit ClpP	C0965_002280	UVZ02675.1	
Clp protease ClpP	C0965_008345	UVZ01872.1	
ATP-dependent Clp protease ATP-binding subunit ClpX	C0965_003700	UVZ02918.1	

ATP-dependent Clp protease ATP-binding subunit*	C0965_008845	UVZ01963.1	
ATP-dependent Clp protease ATP-binding subunit ClpL	C0965_000195	UVZ02318.1	
Trigger factor*	C0965_003695	UVZ02917.1	
**Proteins of the antioxidant response**			
Dyp-type peroxidase*	C0965_002520	UVZ02718.1	
Thiol peroxidase*	C0965_002945	UVZ02794.1	
Glutamate--cysteine ligase	C0965_005845	UVZ01437.1	
Glutamate--cysteine ligase*	C0965_008140	UVZ01838.1	
Glutaredoxin-like protein NrdH	C0965_001845	UVZ02590.1	
Thiol reductant ABC exporter subunit CydD*	C0965_001815	UVZ02584.1	
Thiol reductant ABC exporter subunit CydC*	C0965_001820	UVZ02585.1	
Alkyl hydroperoxide reductase subunit C (Peroxiredoxin)*	C0965_002585	UVZ02729.1	
Alkyl hydroperoxide reductase subunit F*	C0965_002590	UVZ02730.1	
Thioredoxin	C0965_003110	UVZ02826.1	
Thioredoxin*	C0965_009430	UVZ02063.1	
Thioredoxin*	C0965_009840	UVZ02135.1	
Thioredoxin family protein*	C0965_007480	UVZ01727.1	
Peptide-methionine (S)-S-oxide reductase MsrA	C0965_004565	UVZ03072.1	
Peptide-methionine (S)-S-oxide reductase MsrA*	C0965_004580	UVZ03075.1	
Peptide-methionine (S)-S-oxide reductase MsrB*	C0965_005225	UVZ03199.1	
Thioredoxin-disulfide reductase*	C0965_002195	UVZ02658.1	
DsbA family protein	C0965_003385	UVZ02857.1	
Ferrochelatase*	C0965_004110	UVZ02990.1	

DNA starvation/stationary phase protection protein Dps*	C0965_008170	UVZ01843.1	
OsmC family protein (Peroxiredoxin)*	C0965_009955	UVZ02154.1	
Peroxide stress protein YaaA*	C0965_006660	UVZ01575.1	
Copper-translocating P-type ATPase*	C0965_000865	UVZ02432.1	
Copper-translocating P-type ATPase*	C0965_010365	UVZ02222.1	
CopY/TcrY family copper transport repressor*	C0965_006045	UVZ01468	
CopY/TcrY family copper transport repressor	C0965_010370	UVZ02223.1	
Cupredoxin domain-containing protein	C0965_000855	UVZ02430.1	
Cupredoxin domain-containing protein*	C0965_000860	UVZ02431.1	
Multicopper oxidase domain-containing protein*	C0965_008230	UVZ01853.1	
Multicopper oxidase domain-containing protein*	C0965_010770	UVZ03316.1	
Heavy metal transporter ATPase*	C0965_009910	UVZ02145.1	
Heavy metal transporter ATPase	C0965_008510	UVZ01900.1	
Heavy metal-binding domain-containing protein*	C0965_009535	UVZ02077.1	
**Other stress response proteins**			
Alkaline shock response membrane anchor protein AmaP*	C0965_004885	UVZ03131.1	
GlsB/YeaQ/YmgE family stress response membrane protein	C0965_004900	UVZ03134.1	
Glutamate/gamma-aminobutyrate family transporter YjeM	C0965_009245	UVZ02029.1	
Glutamate/gamma-aminobutyrate family transporter YjeM*	C0965_010150	UVZ02188.1	
Translational GTPase TypA*	C0965_003605	UVZ02899.1	
Peroxide stress protein YaaA*	C0965_006660	UVZ01575.1	

Asp23/Gls24 family envelope stress response<br><br>protein*	C0965_004895	UVZ03133.1	
Asp23/Gls24 family envelope stress response protein*	C0965_007075	UVZ01649.1	
Asp23/Gls24 family envelope stress response<br><br>protein*	C0965_007185	UVZ01671.1	
Universal stress protein*	C0965_002825	UVZ02772.1	
Universal stress protein*	C0965_007615	UVZ01753.1	
Universal stress protein*	C0965_009010	UVZ01989.1	
Universal stress protein*	C0965_009985	UVZ02160.1	
Universal stress protein*	C0965_010240	UVZ02206.1	
Universal stress protein	C0965_010610	UVZ03299.1	
